# *per*-Hydroxylated Prism[*n*]arenes: Supramolecularly Assisted Demethylation of Methoxy-Prism[5]arene

**DOI:** 10.1021/acs.orglett.1c02800

**Published:** 2021-10-11

**Authors:** Rocco Del Regno, Paolo Della Sala, Davide Picariello, Carmen Talotta, Aldo Spinella, Placido Neri, Carmine Gaeta

**Affiliations:** Dipartimento di Chimica e Biologia “A. Zambelli”, Università di Salerno, Via Giovanni Paolo II 132, I-84084 Fisciano (Salerno), Italy

## Abstract

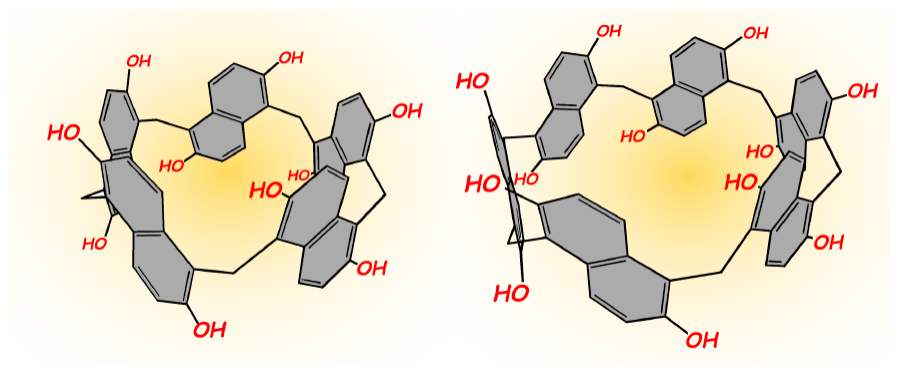

Methoxy-prism[5]arene **PrS[5]**^***Me***^ is demethylated by a supramolecularly assisted reaction. In the presence of a tetramethylammonium cation, **PrS[5]**^***Me***^ is demethylated by BBr_3_ in high yield, while in its absence a 55/40 mixture of **PrS[5]**^***OH***^/**PrS[6]**^***OH***^ is formed. The dealkylation of prismarenes, such as **PrS[6]**^***R***^ (R = Et, *n*Pr) and ***c-*****PrS[5]**^***Me***^, can be easily obtained in high yields in the presence of BBr_3_.

In the past few years, growing interest has been directed toward the synthesis of novel macrocycles^1a^ starting with naphthalene^1b−1e^ or anthracene^2^ monomers. Oxatubarenes,^3^ naphtocages,^4^ zorbarenes,^5^ naphthotubes,^6^ calix[2]naphth[2]arenes,^7^ and prismarenes^8−10^ are naphthalene-based emerging macrocycles, which are of great interest in supramolecular chemistry due to their peculiar structural properties and π-rich deep cavities. Recently, our group reported the prism[*n*]arenes^8−10^ (**PrS[*****n*****]**^***R***^, *n* = 5 and 6, R = Me, Et, *n*Pr, Figure 1), a novel class of macrocyclic hosts constituted by 1,5-methylene-bridged naphthalene units, obtained by one-pot condensation of 2,6-dimethoxynaphthalene and paraformaldehyde in the presence of TFA.^11^ Very recently^12^ the saucer[*n*]arene macrocycles were reported by the one-pot condensation of 2,7-dimethoxynaphthalene and paraformaldehyde.^12^

**Figure 1:**
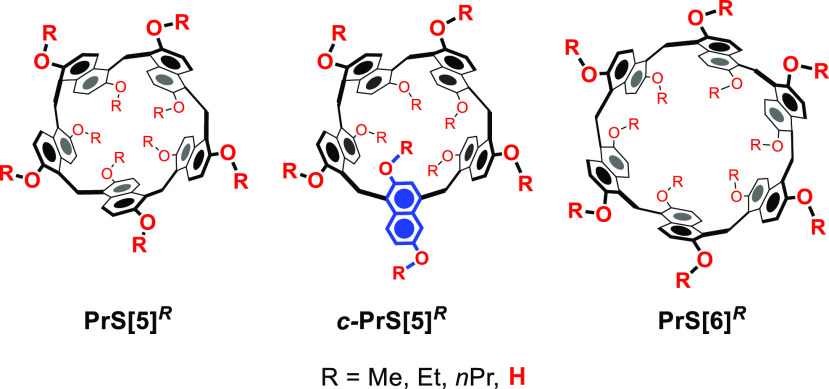
Prism[*n*]arene macrocycles.

Phenol and resorcinol-based macrocycles, such as calixarenes,^13^ resorcinarenes,^14^ and pillararenes,^15^ have become highly studied and attractive synthetic hosts in supramolecular chemistry because of their easy functionalization. In particular, the procedure of functionalization of pillararenes starts primarily by the dealkylation of alkoxy-substituted (−OR) aromatic units.^16^ In fact, *per*-hydroxylated macrocycles are considered as useful synthetic precursors to obtain chemically modified hosts with novel supramolecular properties with respect to the parent macrocycles.^16,17^ Based on these considerations, and with the aim to expand the potentialities of prismarene macrocycles as supramolecular hosts, we decided to investigate a convenient procedure for the synthesis of *per*-hydroxylated prism[*n*]arenes **PrS[*****n*****]**^***OH***^ (*n* = 5 and 6, Figure 1).

Initially, we investigated the demethylation of **PrS[5]**^***Me***^ under the conditions previously reported^15^ for methoxy-pillar[5]arene. Thus, **PrS[5]**^***Me***^ was treated with BBr_3_ in dry CH_2_Cl_2_ at 25 °C for 12 h. The FT ICR MALDI mass spectrum of the crude product of this reaction unambiguously revealed the presence of a mixture of *per*-hydroxylated **PrS[5]**^***OH***^ and **PrS[6]**^***OH***^ derivatives. **PrS[6]**^***OH***^ was separated in 40% yield from **PrS[5]**^***OH***^ (55%) by precipitation from ethyl acetate.

With this result in hand, we attempted the demethylation of **PrS[5]**^***Me***^ by lowering the reaction temperature. Thus, **PrS[5]**^***Me***^ was reacted with BBr_3_ under the conditions reported in Scheme 1, at −78 °C for 1 h and 0 °C for 3 h, but analogously, a mixture of *per*-hydroxylated **PrS[5]**^***OH***^ (55%) and **PrS[6]**^***OH***^ (40%) derivatives was obtained. Chromatographic analysis and HR FT ICR mass spectrometry evidenced that, during the demethylation of **PrS[5]**^***Me***^ in the presence of BBr_3_, the hexamer **PrS[6]**^***Me***^ started to appear after 15 min through the formation of linear oligomers (Figure 2), and successively the mixture of **PrS[5]**^***Me***^ and **PrS[6]**^***Me***^ was demethylated. This is not a surprising result for us; in fact, previously^8,10^ we reported examples of conversion processes between pentamer and hexamer, upon treatment with trifluoracetic acid (TFA) at 70 °C in 1,2-dichloroethane (1,2-DCE) as solvent, in which the conversions occurred by an acid-catalyzed ring opening and macrocyclization mechanism.^8,10^ With the aim to study this conversion under the conditions of solvent and temperature reported in Scheme 1, **PrS[5]**^***Me***^ was treated in CH_2_Cl_2_ at −78 °C for 1 h at 0 °C and for 3 h in the presence of TFA. ^1^H NMR analysis of the crude product revealed the presence of **PrS[6]**^***Me***^ and **PrS[5]**^***Me***^ in a 1:1 ratio (Figure S16).^18^ On the basis of these results, we can assume that, under the conditions reported in Scheme 1, **PrS[5]**^***Me***^ was first converted to **PrS[6]**^***Me***^ (Figure 2) and finally, the mixture of pentamer and hexamer was demethylated.^19^ In other words, the conversion of **PrS[5]**^***Me***^ to **PrS[6]**^***Me***^ is kinetically favored with respect to its dealkylation.^19^ To confirm the role of BBr_3_ in promoting the interconversion in Figure 2, we observed that, in its absence, **PrS[5]**^***Me***^ was stable under the conditions of demethylation in Scheme 1.

**Scheme 1:**
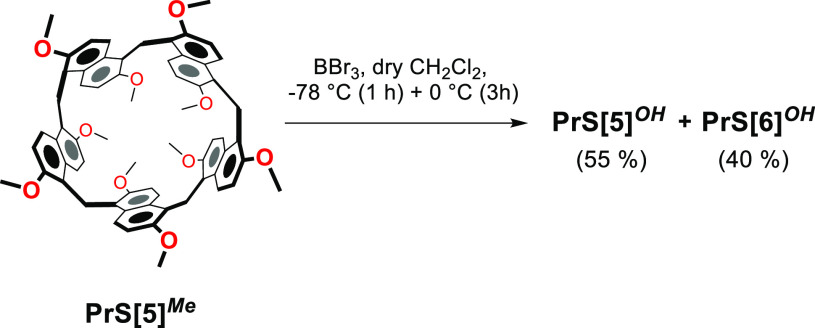
Dealkylation of **PrS[5]**^***Me***^

**Figure 2:**
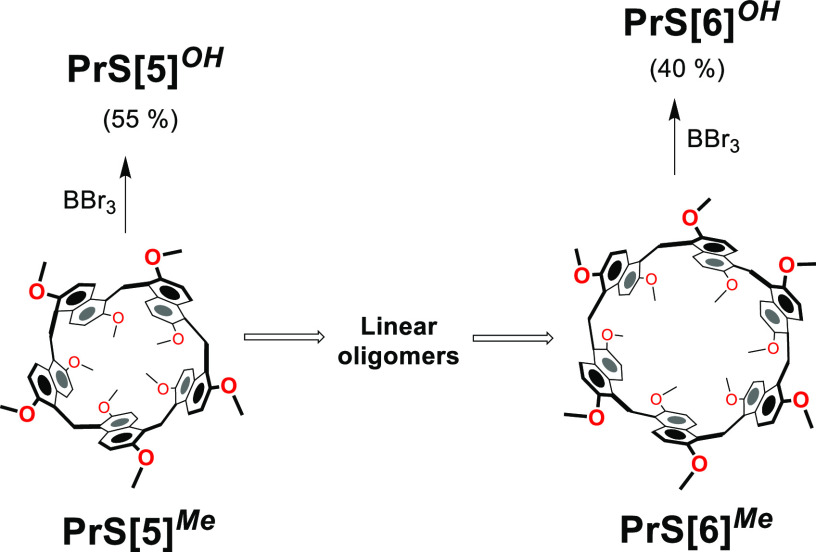
Proposed mechanism for the conversion of **PrS[5]**^***Me***^ to **PrS[6]**^***Me***^ and their demethylation. Reaction conditions in Scheme 1: BBr_3_, *dry* CH_2_Cl_2_, 1 h at −78 °C and 3 h at 0 °C.

At this point we studied the stability of **PrS[5]**^***OH***^ under acid conditions. Interestingly, *per*-hydroxylated **PrS[5]**^***OH***^ was stable up to 24 h in the presence of TFA in 1,2-DCE as solvent (at 70 °C), while polymeric insoluble products were formed in the presence of BBr_3_ in dichloromethane (1 h at −78 °C and 3 h at 0 °C). In both instances, no hint of hexamer **PrS[6]**^***OH***^ was detected in the reaction mixtures, and this result clearly indicated that the conversion of pentamer to hexamer in Figure 2 occurred in the presence of BBr_3_, through the methylated macrocycle, while *per*-hydroxylated **PrS[5]**^***OH***^ did not convert to hexamer **PrS[6]**^***OH***^.

As known,^8,10^ under acid equilibrium conditions the distribution of prismarene macrocycles (pentamer or hexamer) is influenced by the presence of ammonium guests: in the literature this process is named, in a general way, as thermodynamic template effect.^20^

In detail, starting with 2,6-dimethoxynaphthalene and paraformaldehyde, in the presence of TFA in 1,2-DCE as solvent at 70 °C, ***c-*****PrS[5]**^***Me***^ (Figure 1) was obtained in 40% yield (thermodynamic adduct), while its *D*_5_-isomer **PrS[5]**^***Me***^ and hexamer **PrS[6]**^***Me***^ were isolated from the equilibrium mixture by adding as template **1**^+^ and **4**^+^, respectively.^8^ An analogous effect was observed by Chen and co-workers with saucerarenes; in fact, by adding 1,1-dimethylpiperidin-1-ium as the template, saucer[4]arene was selectively obtained.^12^

On this basis, we envisioned studying the demethylation of **PrS[5]**^***Me***^ in the presence of ^+^N(Me)_4_ cation **1**^+^.^8^ In particular, we speculated that the formation of the **1**^+^@**PrS[5]**^***Me***^ complex can affect the **PrS[5]**^***Me***^/**PrS[6]**^***Me***^ ratio and the successive demethylation with BBr_3_.^8^ Thus, **PrS[5]**^***Me***^ was treated (Scheme 2) with BBr_3_ in dry CH_2_Cl_2_ (1 h at −78 °C and 3 h at 0 °C) in the presence of **1**^+^ as iodide salt (1 equiv), and under these conditions **PrS[5]**^***OH***^ was selectively obtained in 90% yield. This result is particularly surprising; in fact, the presence of **1**^+^ strongly improves the selectivity of the demethylation reaction of **PrS[5]**^***Me***^. Probably the formation of **1**^**+**^@**PrS[5]**^**Me**^ complex kinetically favors the demethylation of **PrS[5]**^**Me**^ with respect to its conversion to **PrS[6]**^***Me***^. In fact, as previously reported by us,^10^ upon inclusion of the guest **1**^**+**^, the **PrS[5]**^**Me**^ macrocycle adopts an open conformation, in which the methoxy groups are sterically more accessible with respect to the closed conformation^10^ of **PrS[5]**^**Me**^ in the free state (Figure S20). These results clearly indicated that the demethylation of **PrS[5]**^***Me***^ is supramolecularly driven by the tetramethylammonium guest, which forms the complex **1**^+^@**PrS[5]**^***Me***^ that is easily demethylated.^21^

**Scheme 2:**
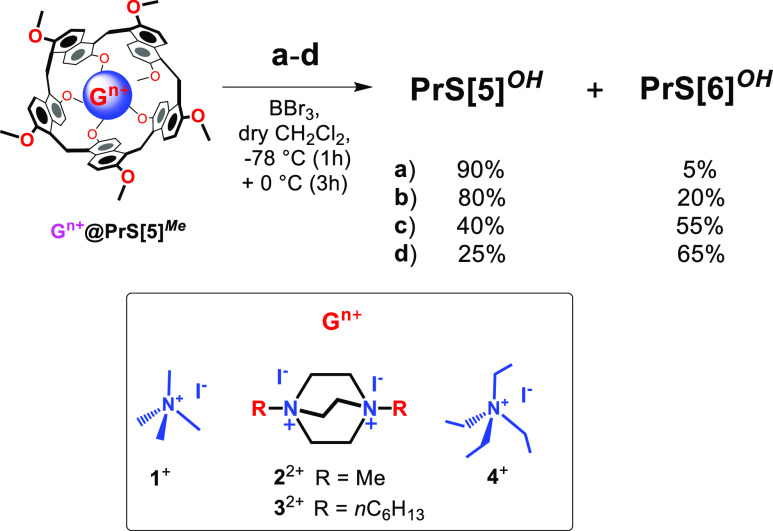
Supramolecularly Assisted Dealkylation of **PrS[5]**^***Me***^ in the Presence of Template Agent **1**^+^–**4**^+^ as Iodide Salts Reaction conditions: BBr_3_, dry CH_2_Cl_2_, 1 h at −78 °C and 3 h at 0 °C: (a) **1**^+^; (b) **2**^2+^; (c) **3**^2+^; (d) **4**^+^.

With these results in hand we attempted the demethylation of **PrS[5]**^***Me***^ in the presence of 1,4-dihexyl-DABCO **3**^2+^, as iodide salt (Scheme 2). As previously reported,^8^ the **3**^2+^**@PrS[5]**^***Me***^ complex shows an association constant value of 3.9 × 10^7^ M^–1^ which is significatively higher than that for **1**^**+**^**@PrS[5]**^***Me***^ (6.4 × 10^4^ M^–1^). When **PrS[5]**^***Me***^ was reacted with BBr_3_ in dry CH_2_Cl_2_ (1 h at −78 °C and 3 h at 0 °C) in the presence of **3**^2+^ as iodide salt (1 equiv), the hexamer **PrS[6]**^***OH***^ was obtained in 55% yield, while the pentamer **PrS[5]**^***OH***^ was obtained in 40% yield (Scheme 2). This result clearly indicates that in the presence of **3**^2+^**@PrS[5]**^***Me***^ complex the demethylation is kinetically underdog with respect to the conversion of **PrS[5]**^***Me***^ to **PrS[6]**^***Me***^, probably due to steric reasons. In fact, inspection of a **3**^2+^**@PrS[5]**^***Me***^ model (Figure S21) suggests that the two methoxy-rims are hindered by the presence of hexyl chains.^8^ To confirm this assumption, **PrS[5]**^***OH***^ was again the favored product (80%, Scheme 2) when the demethylation was performed in the presence of 1,4-dimethyl-DABCO **2**^2+^ bearing less encumbering methyl groups.

Among the ammonium guests so far explored for the *endo*-cavity complexation with prismarene hosts,^8,10^ tetraethylammonium **4**^+^ shows the higher **PrS[6]**^***Me***^/**PrS[5]**^***Me***^ selectivity ratio (S = *K*_**2**__@[**6]**_/*K*_**2**__@[**5]**_= 2700/90 = 30).^8^ For this reason, we studied the demethylation of **PrS[5]**^***Me***^ in the presence of tetraethylammonium **4**^+^ (Scheme 2), which shows low affinity for **PrS[5]**^***Me***^.^8^ When the demethylation reaction of **PrS[5]**^***Me***^ was performed with BBr_3_ in dry CH_2_Cl_2_ (Scheme 2) in the presence of **4**^+^ as iodide salt, **PrS[6]**^***OH***^ was favored (65%) over **PrS[5]**^***OH***^ (25%). Under these conditions (**d** in Scheme 2), initially the conversion of **PrS[5]**^***Me***^ in **PrS[6]**^***Me***^ was kinetically favored with respect to its demethylation. Then the **4**^+^**@PrS[6]**^***Me***^ complex was formed and quickly demethylated.

Considering these results, the question arises as to whether **PrS[6]**^***Me***^ itself can be easily demethylated: in other words, does demethylation of **PrS[6]**^***Me***^ lead to the formation of a mixture of **PrS[6]**^***OH***^/**PrS[5]**^***OH***^? To investigate this aspect, **PrS[6]**^***Me***^ was reacted with BBr_3_ in dry CH_2_Cl_2_ (Scheme 3, 1 h at −78 °C and 3 h at 0 °C), and **PrS[6]**^***OH***^ was obtained in 93% yield. No hint of **PrS[5]**^***OH***^ was detected in the reaction mixture, and this result suggests that no conversion of **PrS[6]**^***Me***^ to **PrS[5]**^***Me***^ occurs. In order to confirm this assumption, **PrS[6]**^***Me***^ was treated with TFA under the usual conditions of solvent and temperature reported in Scheme 3. Thin layer chromatography and ^1^H NMR analysis (Figure S18) clearly indicated the absence of **PrS[5]**^***Me***^, while confused **c-PrS[5]**^**Me**^ began to appear by prolonging the reaction time.^22^ In conclusion, in the presence of TFA in CH_2_Cl_2_, **PrS[5]**^***Me***^ is first converted to **PrS[6]**^***Me***^ and, in the long-run, to ***c*****-PrS[5]**^***Me***^,^8^ while when starting with **PrS[6]**^***Me***^ its slow conversion to ***c-*****PrS[5]**^***Me***^ occurs (Figure S19), and no **PrS[5]**^***Me***^ is formed. These results are in full accord with the data previously reported by us^8^ that indicated **PrS[6]**^***Me***^ as a kinetic intermediate and ***c*****-PrS[5]**^***Me***^ as the thermodynamic macrocycle (Figure 1). Accordingly, when ***c*****-PrS[5]**^***Me***^ was demethylated in the presence of BBr_3_ in CH_2_Cl_2_, ***c*****-PrS[5]**^***OH***^ was formed in 93% yield and no conversion to other prismarenes was observed.

**Scheme 3:**
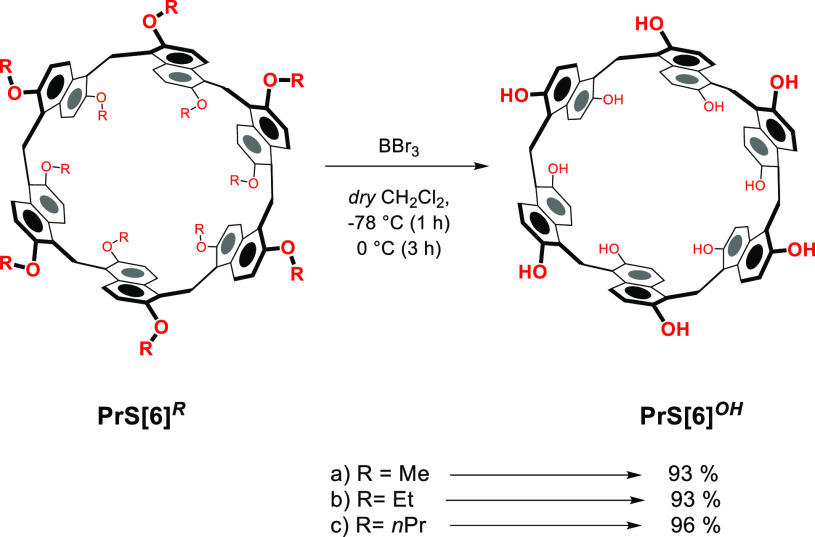
Dealkylation of **PrS[6]**^***R***^

With these results in hand, we attempted the synthesis of *per*-hydroxylated prismarenes starting with ethoxy **PrS[*****n*****]**^***Et***^ and propoxy **PrS[*****n*****]**^***nPr***^ derivatives. As previously reported, **PrS[6]**^***Et***^ and **PrS[6]**^***nPr***^ are obtained in high yields,^10^ and could be very convenient to investigate a procedure of dealkylation starting with these derivatives. When **PrS[6]**^***Et***^ and **PrS[6]**^***nPr***^ were dealkylated in the presence of BBr_3_ in CH_2_Cl_2_, under the conditions reported in Scheme 3, *per*-hydroxylated **PrS[6]**^***OH***^ was selectively obtained in 93% and 96% yield, respectively. The selectivity observed in the dealkylation reactions of **PrS[6]**^***Et***^ and **PrS[6]**^***nPr***^ in the presence of BBr_3_ can be reasonably explained on the basis of their thermodynamic stability in solution under acid conditions.^8,10^ As previously shown,^10^ the crucial factor which determines the stability of ethoxy- and propoxy-prismarenes under acid conditions is the self-filling of their cavity by intramolecular effects of the alkyl chains.^10^ Consequently, in the presence of BBr_3_ in CH_2_Cl_2_, **PrS[6]**^***Et***^ and **PrS[6]**^***nPr***^ were efficiently dealkylated and no hint of other cyclooligomers was present in the reaction mixture.

Differently, when **PrS[5]**^***Et***^ and **PrS[5]**^***nPr***^ were treated with BBr_3_ in CH_2_Cl_2_, the hexamer **PrS[6]**^***OH***^ was obtained in very high yield (90%), while **PrS[5]**^***OH***^ was detected in very low yield (<10%), to confirm that, under acid conditions in the presence of BBr_3_, **PrS[5]**^***Et***^ and **PrS[5]**^***nPr***^ convert to the more stable hexamers. Clearly, under these conditions the conversion of pentamers **PrS[5]**^***Et***^ and **PrS[5]**^***nPr***^ to their respective hexamers is kinetically favored with respect to dealkylation of ethyl and propyl chains.

When the dealkylation of **PrS[5]**^***Et***^ with BBr_3_ was performed in the presence of **1**^+^ as iodide salt, then the pentamer **PrS[5]**^***OH***^ was detected in 24% yield, while **PrS[6]**^***OH***^ was formed in 69% yield. This result confirms that the formation of the **1**^+^**@PrS[5]**^***Et***^ complex accelerates the dealkylation with respect to free **PrS[5]**^***Et***^.

In conclusion, here is described the supramolecularly assisted dealkylation of methoxy-prism[5]arene **PrS[5]**^***Me***^. The conversion of the pentamer **PrS[5]**^***Me***^ to hexamer is kinetically favored with respect to the demethylation. Differently, when the complex **1**^+^**@PrS[5]**^***Me***^ is formed then the reaction of demethylation becomes kinetically favored with respect to the conversion pentamer → hexamer. The dealkylation of prismarenes, such as **PrS[6]**^***R***^ (R = Et, *n*Pr) and ***c-*****PrS[5]**^***Me***^, can be easily obtained in high yields in the presence of BBr_3_. The *per*-hydroxylated prismarenes described here can be considered as useful synthetic precursors to obtain novel prismarene hosts with intriguing supramolecular properties. Finally, we believe that the procedure of supramolecularly assisted demethylation described here for prism[5]arene could be useful for other macrocycles.

## Supporting Information Available

Detailed synthetic procedures, 1D and 2D NMR spectra of per-hydroxylated prismarenes, HR mass spectra. (PDF)
