# Supplementary material for: per-Hydroxylated Prism[n]arenes: Supramolecularly Assisted Demethylation of Methoxy-Prism[5]arene
Source: Org Lett. 2021 Oct 11;23(21):8143–6. doi: 10.1021/acs.orglett.1c02800 (PMC8576831; doi:10.1021/acs.orglett.1c02800)
Supplement: Supplementary file 1 — ol1c02800_si_001.pdf [file ol1c02800_si_001.pdf]

Supporting Information

per-Hydroxylated Prism[*n*]arenes: Supramolecularly Assisted Demethylation of Methoxy-Prism[5]arene

*Rocco Del Regno, Paolo Della Sala, Davide Picariello, Carmen Talotta, Aldo Spinella, Placido Neri, Carmine Gaeta**

Dipartimento di Chimica e Biologia "A. Zambelli", Università di Salerno, Via Giovanni Paolo II 132, I-84084 Fisciano (Salerno), Italy.

**Table of Contents**	**Pages**
General Experimental Details	**S2**
General procedures for the synthesis of *per*-hydroxylated Prism[n]arenes (PrS[n]^{OH})	**S2 - S5**
Copies of 1D, 2D NMR and HR Mass Spectra	**S6 – S20**
Conversion of **PrS[5]^{Me}** to **PrS[6]^{Me}** in the presence of TFA	**S21**
Conversion of **PrS[5]^{Me}** to *c*-**PrS[5]^{Me}** in the presence of TFA	**S22**
Reaction of **PrS[6]^{Me}** in the presence of TFA	**S23-S24**
Computational Details	**S25 - S40**
References	**S41**

General Experimental Details

HR MALDI mass spectra were recorded on a Bruker Solarix FT-ICR mass spectrometer equipped with a 7T magnet. The samples recorded in MALDI were prepared by mixing 10 μL of analyte in methanol (1 mg/mL) with 10 μL of solution of 2,5-dihydroxybenzoic acid (10 mg/mL in Methanol). The mass spectra were calibrated externally, and a linear calibration was applied. Dichloromethane was dried by heating under reflux over calcium hydride.[1] All chemicals reagents grade was used without further purification and were used as purchased. Reaction temperatures were measured externally. Reactions were monitored by TLC silica gel plates (0.25 mm) and visualized by UV light 254 nm, or by spraying with H_2SO_4−$Ce(SO_4)_2$. NMR spectra were recorded on a Bruker Avance-600 [600 (^{1}H) and 150 MHz (^{13}C)], Bruker Avance-400 [400 (^{1}H) and 100 MHz (^{13}C)], Avance-300 MHz [300 (^{1}H) and 75 MHz (^{13}C)] or Avance-250 MHz [250 (^{1}H) and 62.5 MHz (^{13}C)] spectrometers. Chemical shifts are reported relative to the residual solvent peak.[2] Standard pulse programs, provided by the manufacturer, were used for 2D COSY-45, 2D HSQC and NOESY experiments.

The derivatives ***c*-PrS[5]**** ^{Me}, **PrS[5]**^{R} and **PrS[6]**^{R} (R = Me, Et, *n*-Pr) were synthesized according to literature procedures.[3,4] The ^{1}H and ^{13}C NMR spectra of ***c*-PrS[5]**^{Me}, **PrS[5]**^{R} and **PrS[6]**^{R} (R = Me, Et, *n*-Pr) are in accord with those reported in literature.[3,4]

General procedures for the synthesis of *per*-hydroxylated Prism[n]arenes (PrS[n]^{OH}).

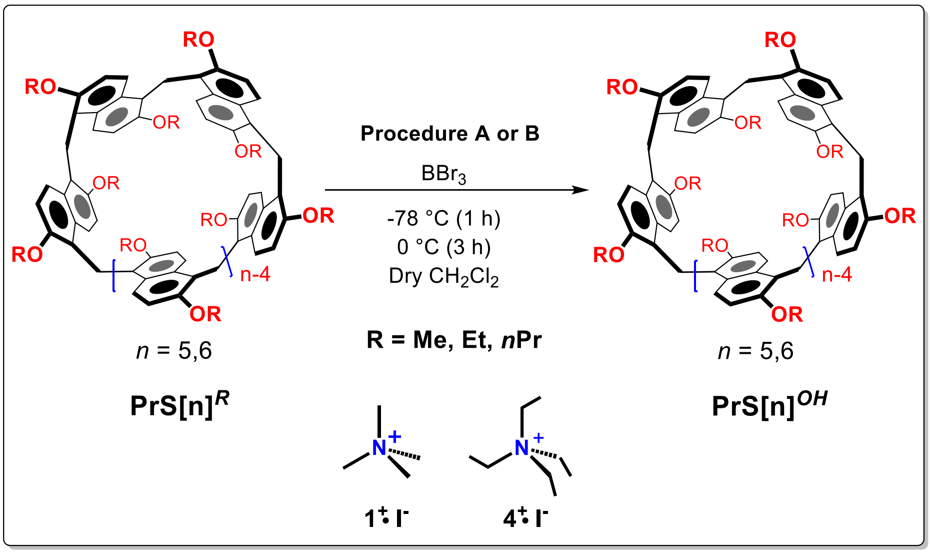

Procedure A: To a solution of **PrS[n]**^{R} in dry CH_2Cl_2 (5.0 mM) was added BBr_3 under nitrogen atmosphere at –78 °C. The mixture was stirred at this temperature for 1 h, and at 0 °C for three hours.

- Starting by ***c*-PrS[5]**^{Me} (200 mg, 0.20 mmol) and BBr_3 (1.9 mL, 20.00 mmol): The reaction was stopped by adding slowly an aqueous solution of $NaHCO_3$ (5.0 g of $NaHCO_3$ dissolved in 50 mL of cold water) and, then, the precipitate was filtered.

The solid was washed with a 10 % aqueous solution of sodium sulfite (50 mL), and two times with water (2 x 50 mL). The precipitate consisted of ***c*-PrS[5]*^{OH}*** in 93 % yield (160 mg), as a white solid.

- Starting by **PrS[5]*^{Me}*** (50 mg, 50.00 µmol) and BBr_3 (0.5 mL, 5.00 mmol): The reaction was stopped by adding slowly an aqueous solution of $NaHCO_3$ (1.3 g of $NaHCO_3$ dissolved in 15 mL of cold water) and, then, the precipitate was filtered. The solid was washed with a 10 % aqueous solution of sodium sulfite (25 mL), and, two times with water (2 x 50 mL). ^{1}H NMR analysis and HR FT ICR MALDI mass spectrum of the crude product indicated that the precipitate was constituted by a mixture of **PrS[5]*^{OH}*** and **PrS[6]*^{OH}***. Ethyl acetate was added (25 mL) and the suspension was stirred at room temperature for 10 min. The precipitate was filtered off to give **PrS[6]*^{OH}*** in 40 % yield (17 mg), while the filtrated contained **PrS[5]*^{OH}*** that was recovered in 55 % yield (24 mg) after evaporation of the solvent under reduced pressure.
- Starting by **PrS[5]*^{Et}*** (50 mg, 43.80 µmol) and BBr_3 (0.4 mL, 4.00 mmol): The reaction was stopped by adding slowly an aqueous solution of $NaHCO_3$ (1.1 g of $NaHCO_3$ dissolved in 15 ml of cold water) and, then, the precipitate was filtered. The solid was washed with a 10 % aqueous solution of sodium sulfite (25 mL), and two times with water (2 x 25 mL). The precipitate consisted of **PrS[6]*^{OH}*** in 90 % yield (34 mg), as a white solid.
- Starting by **PrS[5]*^{nPr}*** (50 mg, 39.00 µmol) and BBr_3 (0.4 mL, 4.00 mmol): The reaction was stopped by adding slowly an aqueous solution of $NaHCO_3$ (1.1 g of $NaHCO_3$ dissolved in 15 mL of cold water) and, then, the precipitate was filtered. The solid was washed with a 10% aqueous solution of sodium sulfite (25 mL), and, later, two times with water (2 x 25 mL). The precipitate consisted of **PrS[6]*^{OH}*** in 90 % yield (30 mg), as a white solid.
- Starting by **PrS[6]*^{Me}*** (100 mg, 83.30 µmol) and BBr_3 (1.0 mL, 9.60 mmol): The reaction was stopped by adding slowly an aqueous solution of $NaHCO_3$ (2.6 g of $NaHCO_3$ dissolved in 50 ml of cold water) and, then, the precipitate was filtered. The solid was washed with a 10% aqueous solution of sodium sulfite (50 mL), and, later, two times with water (2 x 50 mL). The precipitate consisted of **PrS[6]*^{OH}*** in 93 % yield (80 mg), as a white solid.
- Starting by **PrS[6]*^{Et}*** (200 mg, 146.01 µmol) and BBr_3 (1.7 mL, 18.00 mmol): The reaction was stopped by adding slowly an aqueous solution of $NaHCO_3$ (4.4 g of $NaHCO_3$ dissolved in 50 ml of cold water) and, then, the precipitate was filtered. The solid was washed with a 10% aqueous solution of sodium sulfite (50 mL), and, later, two times with water (2 x 50 mL). The precipitate consisted of **PrS[6]*^{OH}*** in 93 % yield (140 mg), as a white solid.
- Starting by **PrS[6]*^{nPr}*** (200 mg, 0.13 mmol) and BBr_3 (1.5 mL, 15.60 mmol): The reaction was stopped by adding slowly an aqueous solution of $NaHCO_3$ (4.6 g of $NaHCO_3$ dissolved in 50 ml of cold water) and, then, the precipitate was filtered. The solid was washed with a 10% aqueous solution of sodium sulfite (50 mL), and, later, two times with water (2 x 50 mL). The precipitate consisted of **PrS[6]*^{OH}*** in 96 % yield (130 mg), as a white solid.

Procedure B: To a solution of **PrS[n]*^{R}*** and templating agent **1^{+}·I^{-}** or **4^{+}·I^{-}** (1 equiv) in dry CH_2Cl_2 (5.0 mM) was added BBr_3 under nitrogen atmosphere at –78 °C. The mixture was stirred at this temperature for 1 h and at 0 °C for three hours.

- Starting by **PrS[5]*^{Me}*** (50 mg, 50.00 µmol), the templating agent **1^{+}·I^{-}** (10 mg, 50.00 µmol) and BBr_3 (0.5 mL, 5.00 mmol): The reaction was stopped by adding slowly an aqueous solution of $NaHCO_3$ (1.3 g of $NaHCO_3$ dissolved in 15 mL of cold water). Then, 30 mL of ethyl acetate were added to the reaction mixture. The aqueous layer was washed with ethyl acetate (3 x 20 mL). Later, the organic layer was washed with a 10 % aqueous solution of sodium sulfite (25 mL), and it was dried with Na_2SO_4 and concentrated to give, as a white solid, the macrocycle **PrS[5]*^{OH}*** in 90 % yield (38 mg).
- Starting by **PrS[5]*^{Et}*** (50 mg, 43.80 µmol), the templating agent **1^{+}·I^{-}** (8.0 mg, 43.80 µmol) and BBr_3 (0.4 mL, 4.00 mmol): The reaction was stopped by adding slowly an aqueous solution of $NaHCO_3$ (1.1 g of $NaHCO_3$ dissolved in 15 mL of cold water) and, then, the precipitate was filtered. The solid was washed with a 10 % aqueous solution of sodium sulfite (25 mL), and, later, two times with water (2 x 25 mL). The precipitate was filtered. Ethyl acetate was added (25 mL) and the suspension was stirred at room temperature for 10 min. The precipitate was filtered off to give and **PrS[6]*^{OH}*** in 69 % yield (26 mg), while the filtrated contained **PrS[5]*^{OH}*** that was recovered in 24 % yield (9 mg) after evaporation of the solvent under reduced pressure.
- Starting by **PrS[5]*^{nPr}*** (50 mg, 39.00 µmol), the templating agent **1^{+}·I^{-}** (8 mg, 39.00 µmol) and boron bromide (0.4 mL, 4.00 mmol): The reaction was stopped slowly adding an aqueous solution of $NaHCO_3$ (1.1 g of $NaHCO_3$ dissolved in 15 ml of cold water) and, then, the precipitate was filtrated. The solid was washed with a 10% aqueous solution of sodium sulfite (25 mL), and, later, two times with water (2 x 25 mL). The precipitate was recovered. Ethyl acetate was added (25 mL) and the suspension was stirred at room temperature for 10 min. The precipitate was filtered off to give and **PrS[6]*^{OH}*** in 84 % yield (28 mg), while the filtrated contained **PrS[5]*^{OH}*** that was recovered in 9 % yield (3 mg) after evaporation of the solvent under reduced pressure.
- Starting by **PrS[5]*^{Me}*** (50 mg, 50.00 µmol), the templating agent **4^{+}·I^{-}** (13 mg, 50.00 µmol) and BBr_3 (0.5 mL, 5.00 mmol): The reaction was stopped by adding slowly an aqueous solution of $NaHCO_3$ (1.3 g of $NaHCO_3$ dissolved in 15 mL of cold water) and, then, the precipitate was filtered. The solid was washed with a 10% aqueous solution of sodium sulfite (25 mL), and, later, two times with water (2 x 25 mL). The precipitate was recovered. Ethyl acetate was added (25 mL) and the suspension was stirred at room temperature for 10 min. The precipitate was filtered off to give **PrS[6]*^{OH}*** in 65 % yield (28 mg), while the filtrated contained **PrS[5]*^{OH}*** that was recovered in 25 % yield (11 mg) after evaporation of the solvent under reduced pressure.

Derivative *c*-PrS[5]^{OH}:

Mp: > 250 °C dec.

^{1}H NMR (400 MHz, DMSO-d_6, 298 K): δ 9.63 - 9.54 (*overlapped*, ArO*H*, 5H) 9.45 (*s*, ArO*H*, 1H), 9.19 (*s*, ArO*H*, 1H), 9.04 (*s*, ArO*H*, 1H), 8.80 (*d*, *J* = 9.3 Hz, Ar*H*, 1H), 8.65 (*d*, *J* = 9.3 Hz, Ar*H*, 1H), 8.60 (*d*, *J* = 9.4 Hz, Ar*H*, 1H), 8.55 (*d*, *J* = 9.2 Hz, Ar*H*, 1H), 8.36 - 8.33 (*overlapped*, Ar*H* + ArO*H*, 2H), 8.20 (*d*, *J* = 9.1 Hz, Ar*H*, 1H), 8.09 (*s*, ArO*H*, 1H), 8.02 (*overlapped*, Ar*H*, 2H), 7.39 (*d*, *J* = 2.2 Hz, Ar*H*, 1H), 7.07 - 6.78 (*overlapped*, Ar*H*, 9H), , 6.43 (*d*, *J* = 9.1 Hz, Ar*H*, 1H), 5.76 (*s*, Ar*H*, 1H), 4.57 - 4.14 (*overlapped*, ArCH_2Ar, 10H).

^{13}C-NMR (100 MHz, DMSO-d_6, 298 K): δ 151.9, 150.7, 150.3, 149.0, 148.8, 148.7,147.7, 132.0, 129.6, 129.4, 129.0, 128.7, 128.4, 128.0, 127.4, 126.9, 124.9, 124.7, 124.3, 123.4, 122.8, 121.3, 120.4, 120.1, 119.8, 119.0, 118.5, 117.7, 117.5, 117.3, 116.9, 116.7, 104.6, 25.7, 21.6, 20.8, 20.4, 20.2.

HRMS (FT-ICR MALDI) m/z $[M]^+$ calcd for $C_{55}H_{40}O_{10}$: 860.2622; found: 860.2622.

Derivative PrS[5]^{OH}:

Mp: > 245 °C dec.

^{1}H NMR (600 MHz, CD_3OD, 298 K): δ 7.98 (*br s*, Ar*H*, 10H), 6.76 (*d*, *J* = 9.0 Hz, Ar*H*, 10H), 4.58 (*s*, ArCH_2Ar, 10H).

^{13}C-NMR (100 MHz, CD_3OD, 298 K): δ 150.5, 130.5, 124.8, 121.0, 118.3, 22.0

HRMS (FT-ICR MALDI) m/z $[M]^+$ calcd for $C_{55}H_{40}O_{10}$: 860.2622; found: 860.2623.

Derivative PrS[6]^{OH}:

Mp: > 250 °C dec.

^{1}H NMR (300 MHz, DMSO-d_6, 373 K): *δ* 7.86 (*d*, *J* = 8.7 Hz, Ar*H*, 12H), 6.87 (*d*, *J* = 8.7 Hz, Ar*H*, 12H), 4.53 (*s*, ArCH_2Ar, 12H).

^{13}C-NMR (100 MHz, DMSO-d_6, 298 K): *δ* 148.8, 129.0, 123.3, 120.1, 117.4, 20.8.

HRMS (FT-ICR MALDI) m/z $[M]^+$ calcd for $C_{66}H_{48}O_{12}$: 1032.3146; found: 1032.3148.

Copies of 1D, 2D NMR and HR Mass Spectra

^{1}H NMR, ^{13}C NMR and HR mass spectra of derivative *c*-PrS[5]^{OH}

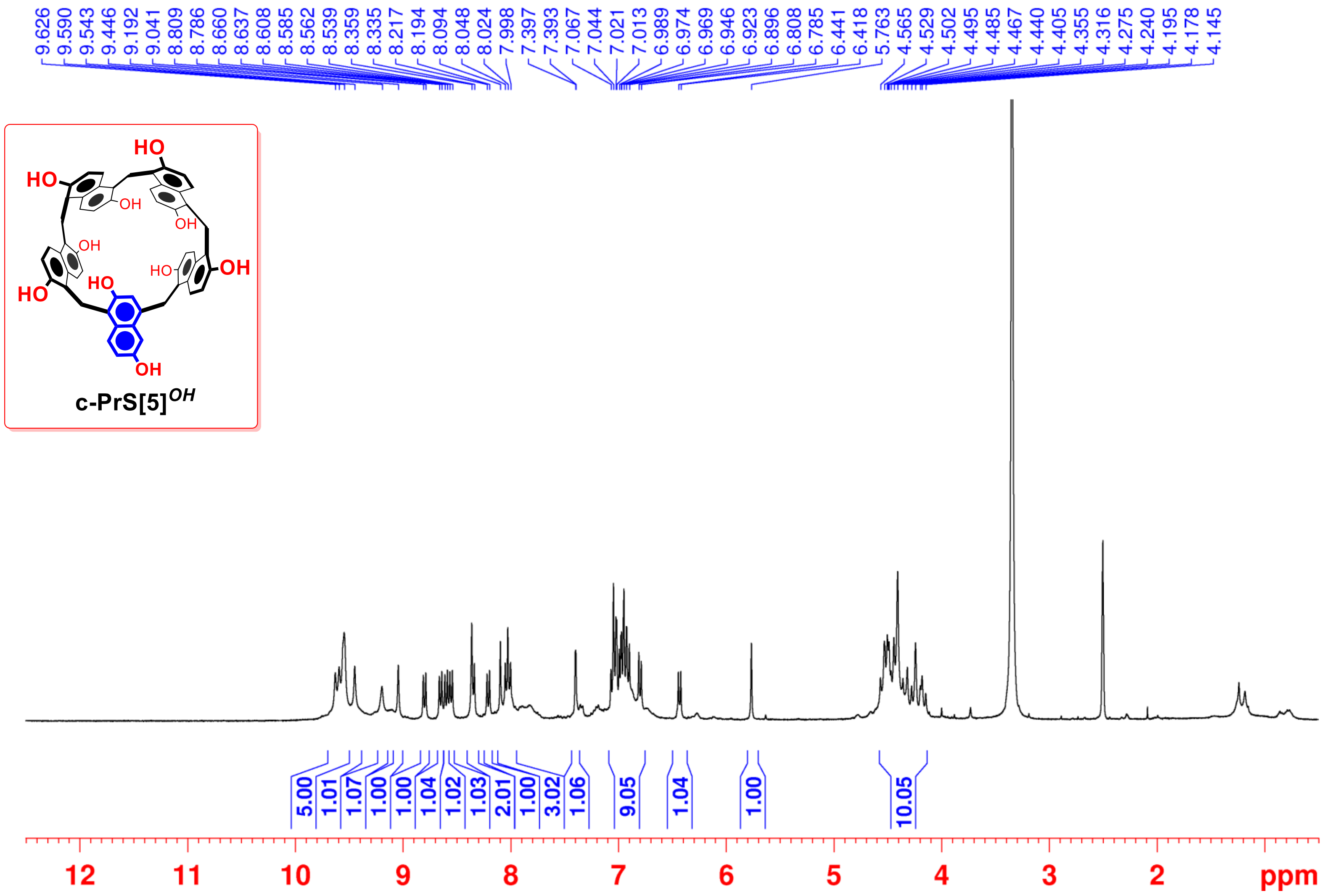

Figure S1: ^{1}H NMR spectrum of ***c*-PrS[5]^{OH}** (DMSO-d_6, 400 MHz, 298 K).

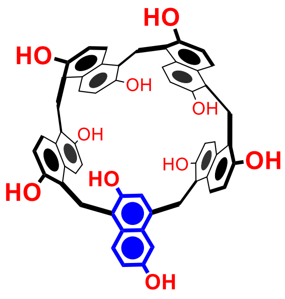

c-PrS[5]^{OH}

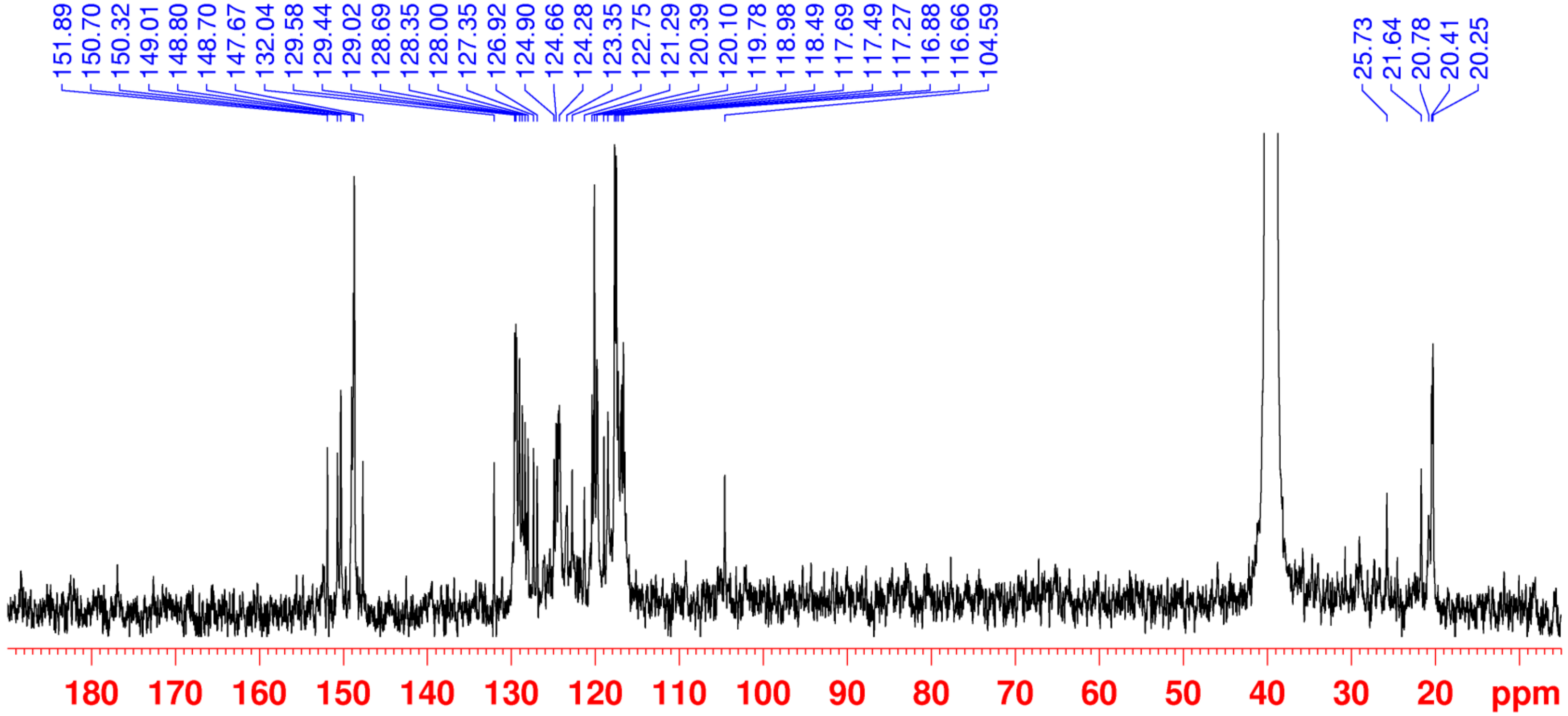

Figure S2: ^{13}C NMR spectrum of ***c*-PrS[5]^{OH}** (DMSO-d_6, 100 MHz, 298 K).

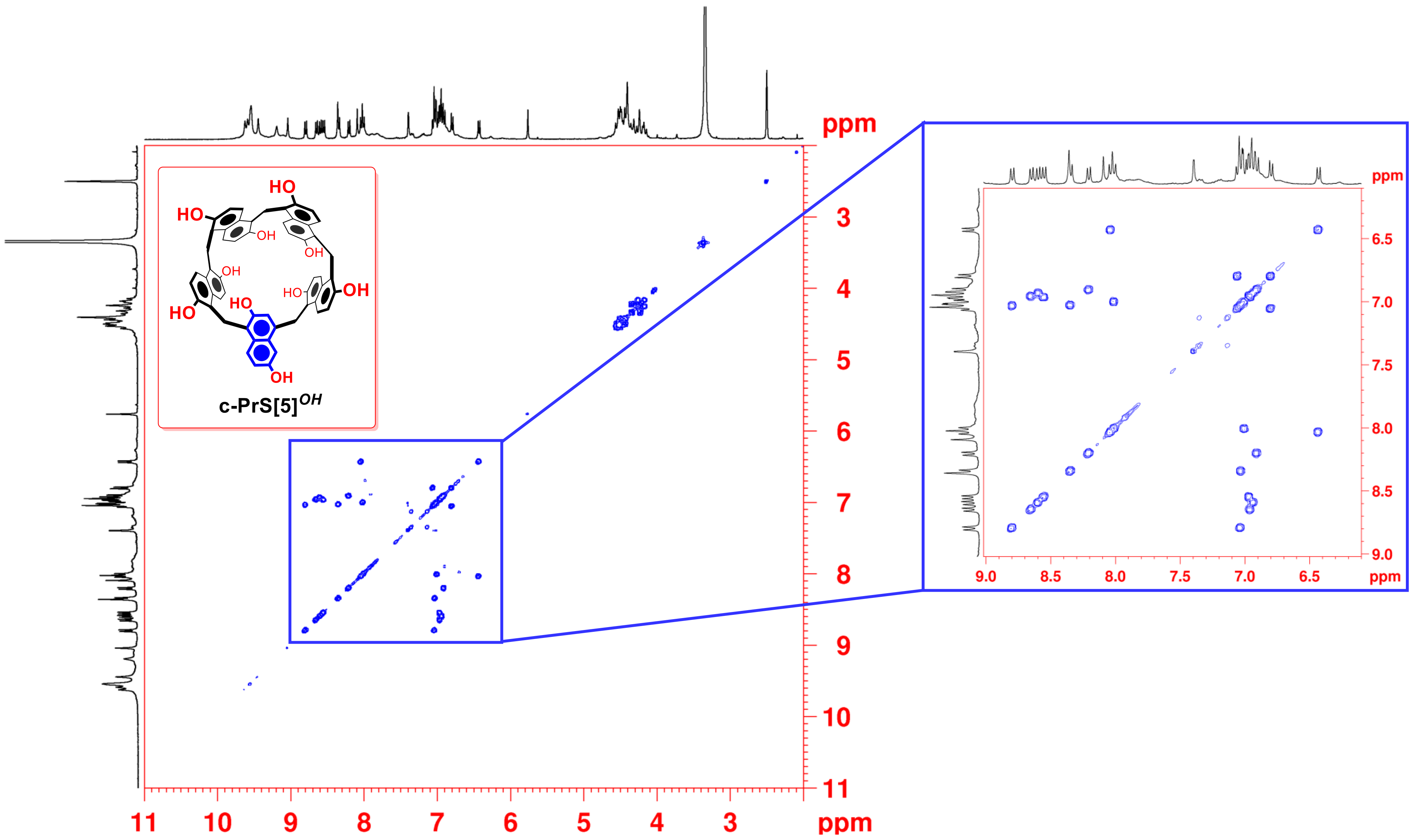

Figure S3: DQF COSY spectrum of ***c*-PrS[5]^{OH}** (DMSO-d_6, 400 MHz, 298 K).

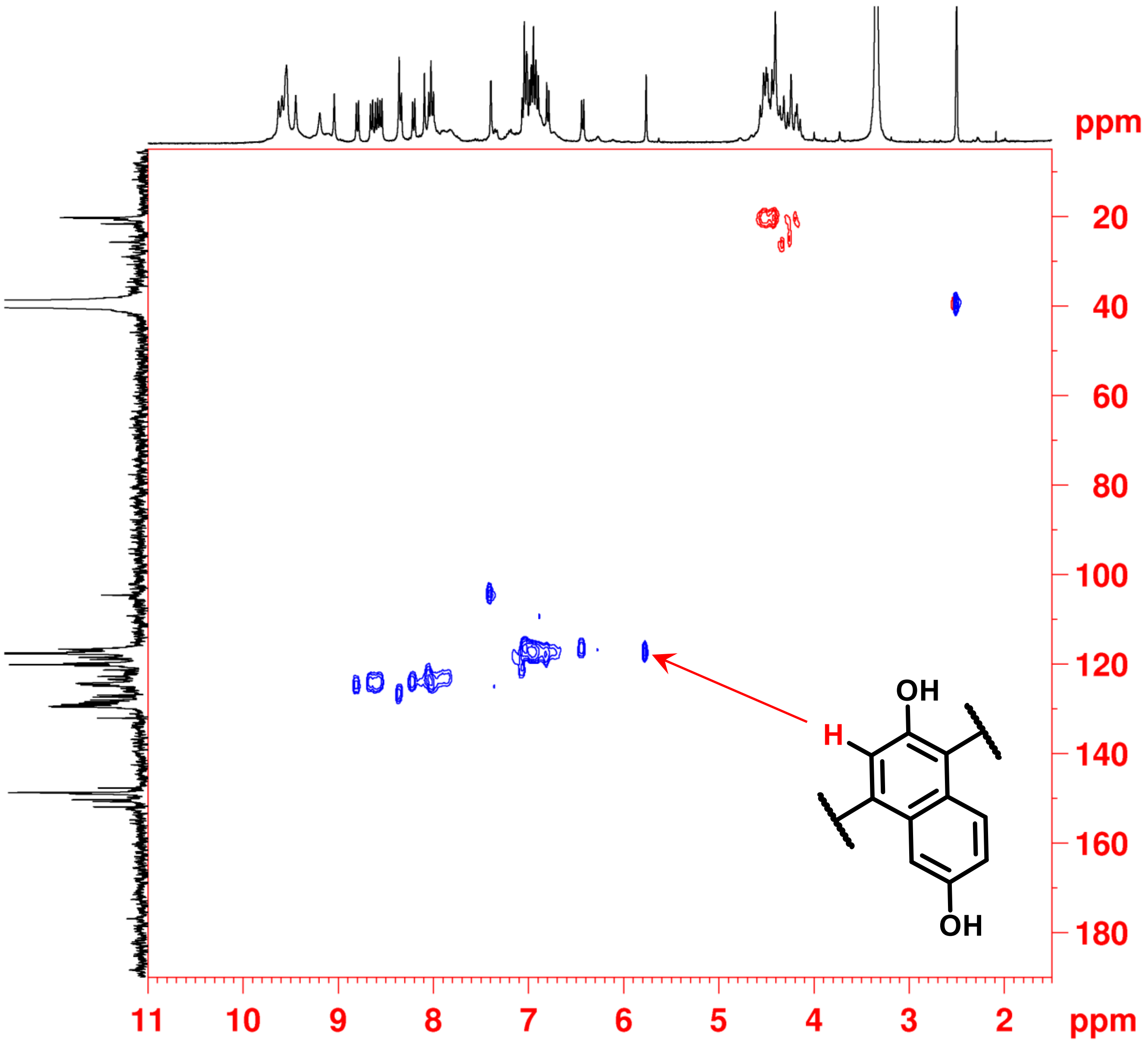

Figure S4: HSQC spectrum of ***c*-PrS[5]*OH*** (DMSO-d_6, 400 MHz, 298 K).

VT NMR studies of derivative *c*-PrS[5]^{OH}

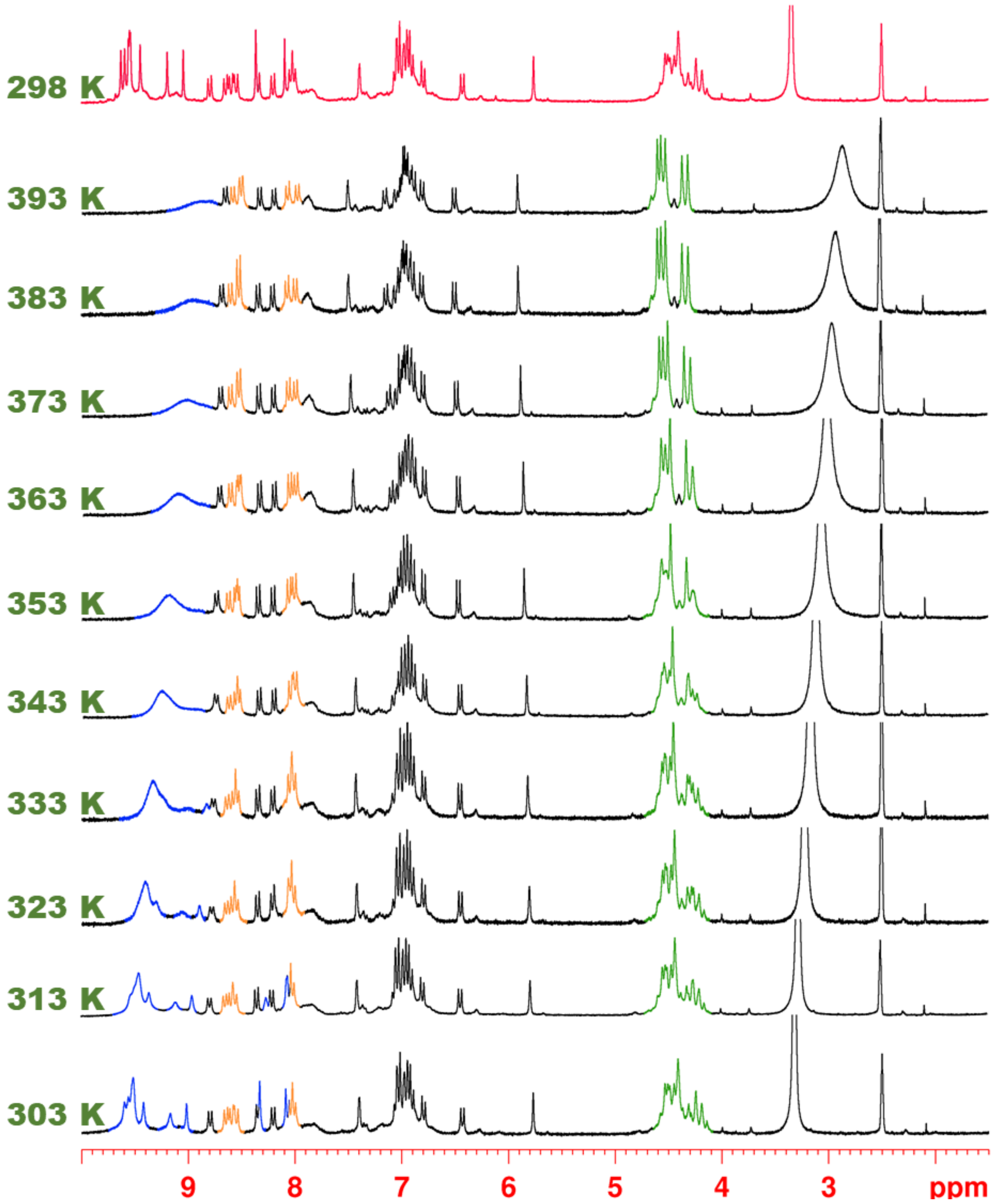

Figure S5: ^{1}H NMR spectrum of ***c*-PrS[5]^{OH}** (DMSO-d_6, 300 MHz) at (from bottom to top): 303, 313, 323, 333, 343, 353, 363, 373, 383 and 393 K; in red the spectrum at 298 K after high temperature acquisition.

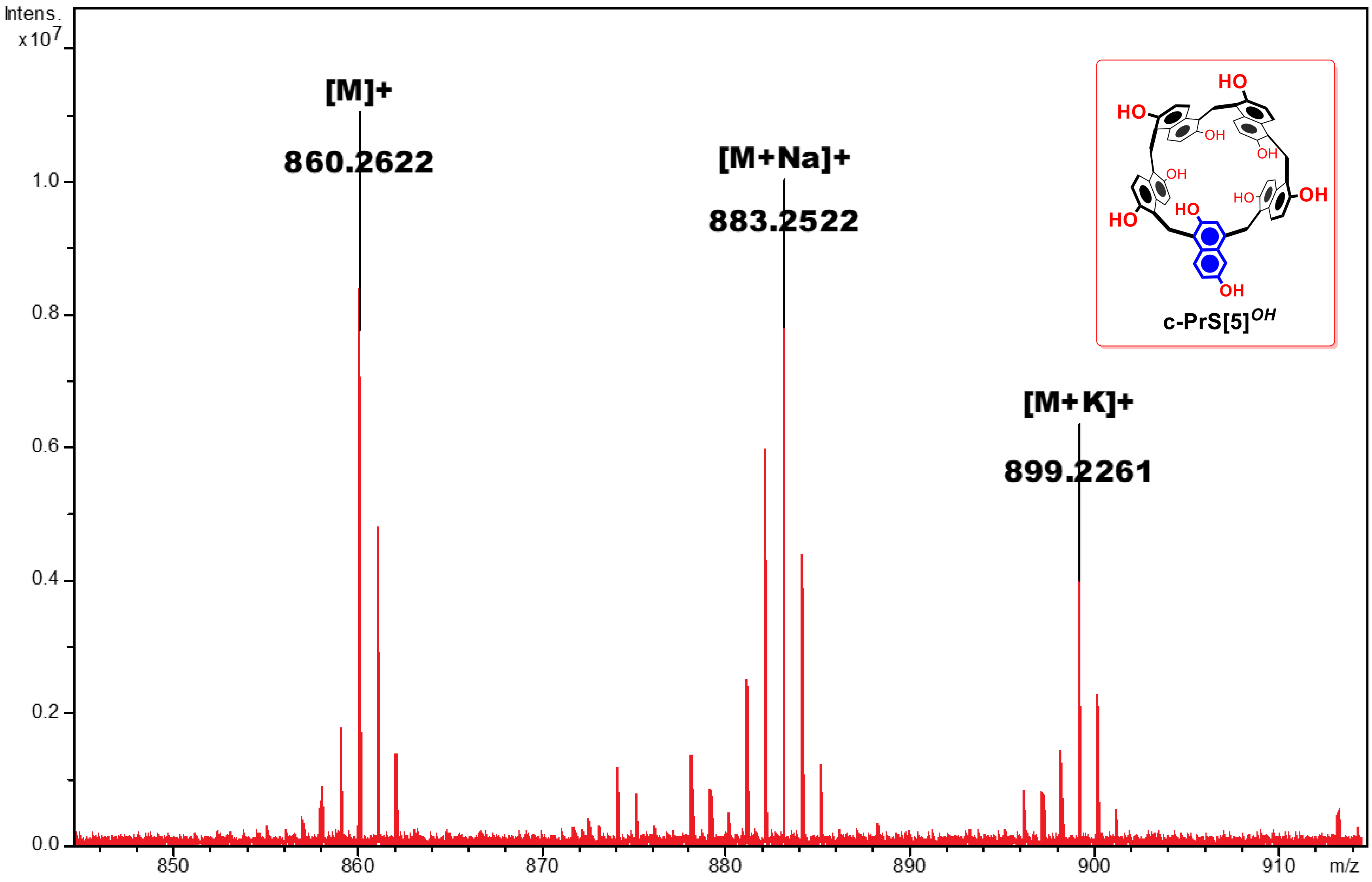

Figure S6: Significant portion of the HR MALDI FT-ICR mass spectrum of ***c*-PrS[5]**^{OH} $[M]^+$, $[M+Na]^+$ and $[M+K]^+$.

1D NMR and HR mass spectra of derivative PrS[5]^{OH}

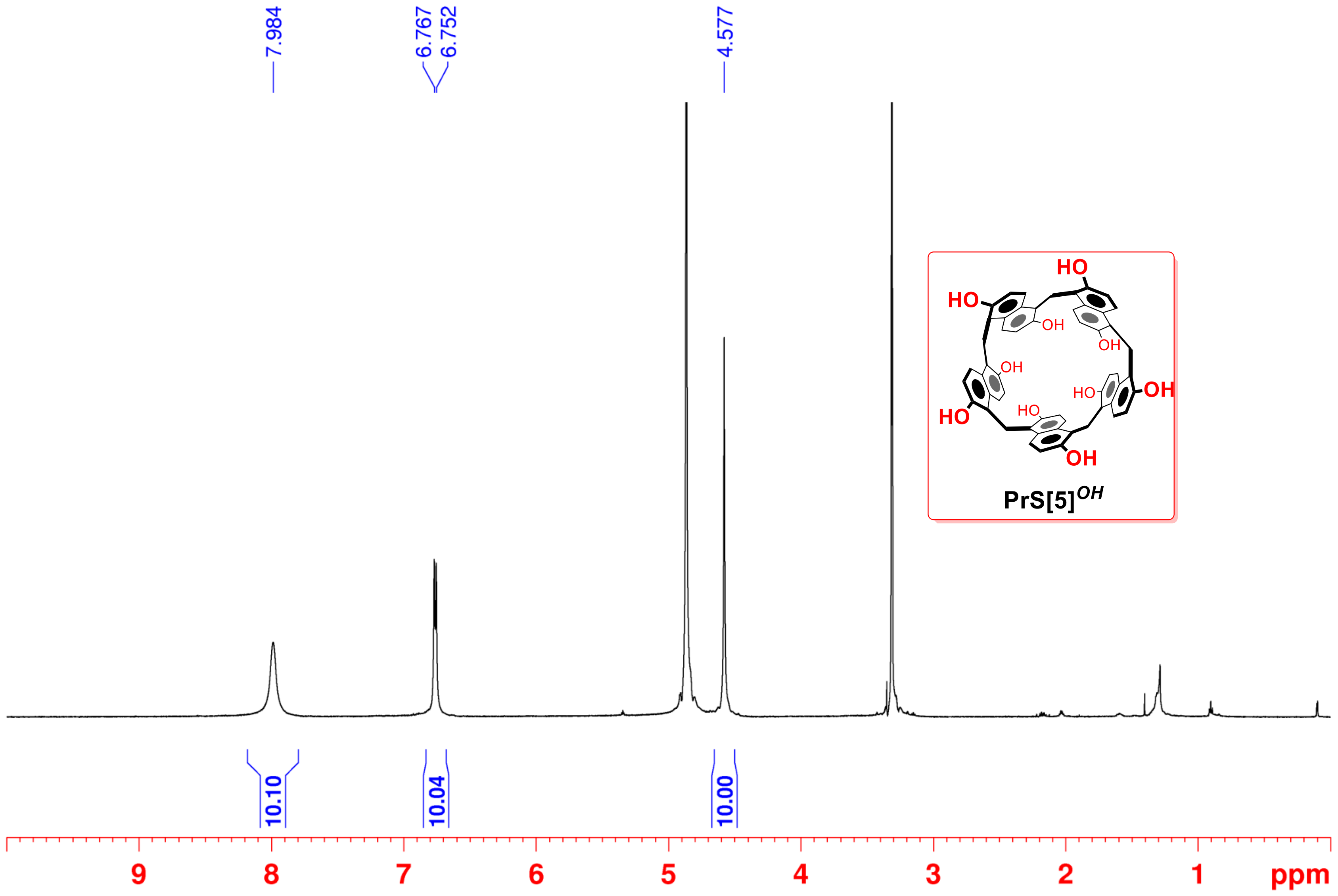

Figure S7: ^{1}H NMR spectrum of **PrS[5]^{OH}** (CD_3OD, 600 MHz, 298 K).

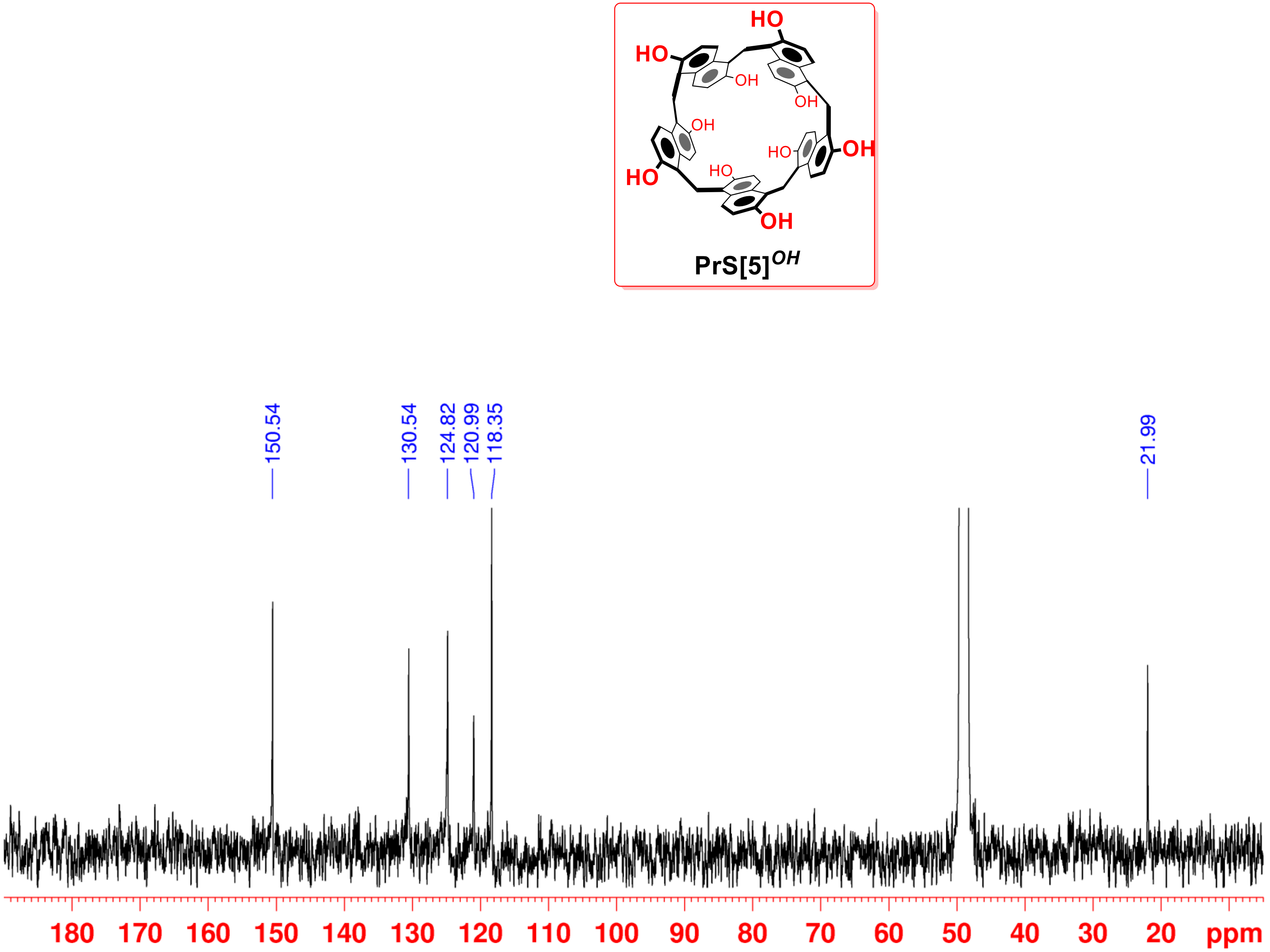

Figure S8: ^{13}C NMR spectrum of **PrS[5]^{OH}** (CD_3OD, 100 MHz, 298 K).

VT NMR studies of derivative PrS[5]^{OH}

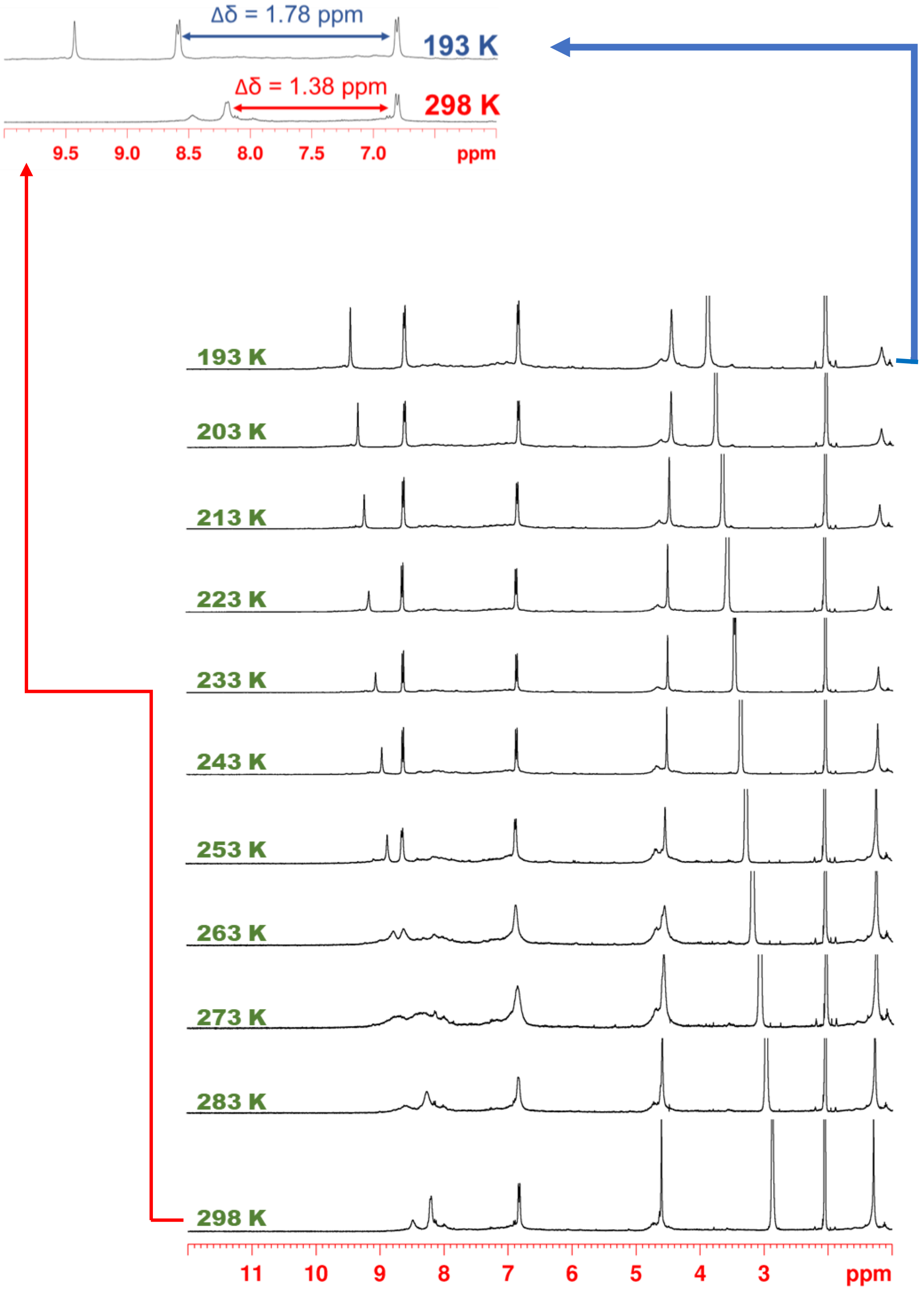

Figure S9: ^{1}H NMR spectrum of **PrS[5]^{OH}** (Acetone-d_6, 400 MHz) at (from bottom to top): 298, 283, 273, 263, 253, 243, 233, 223, 213, 203 and 193 K.

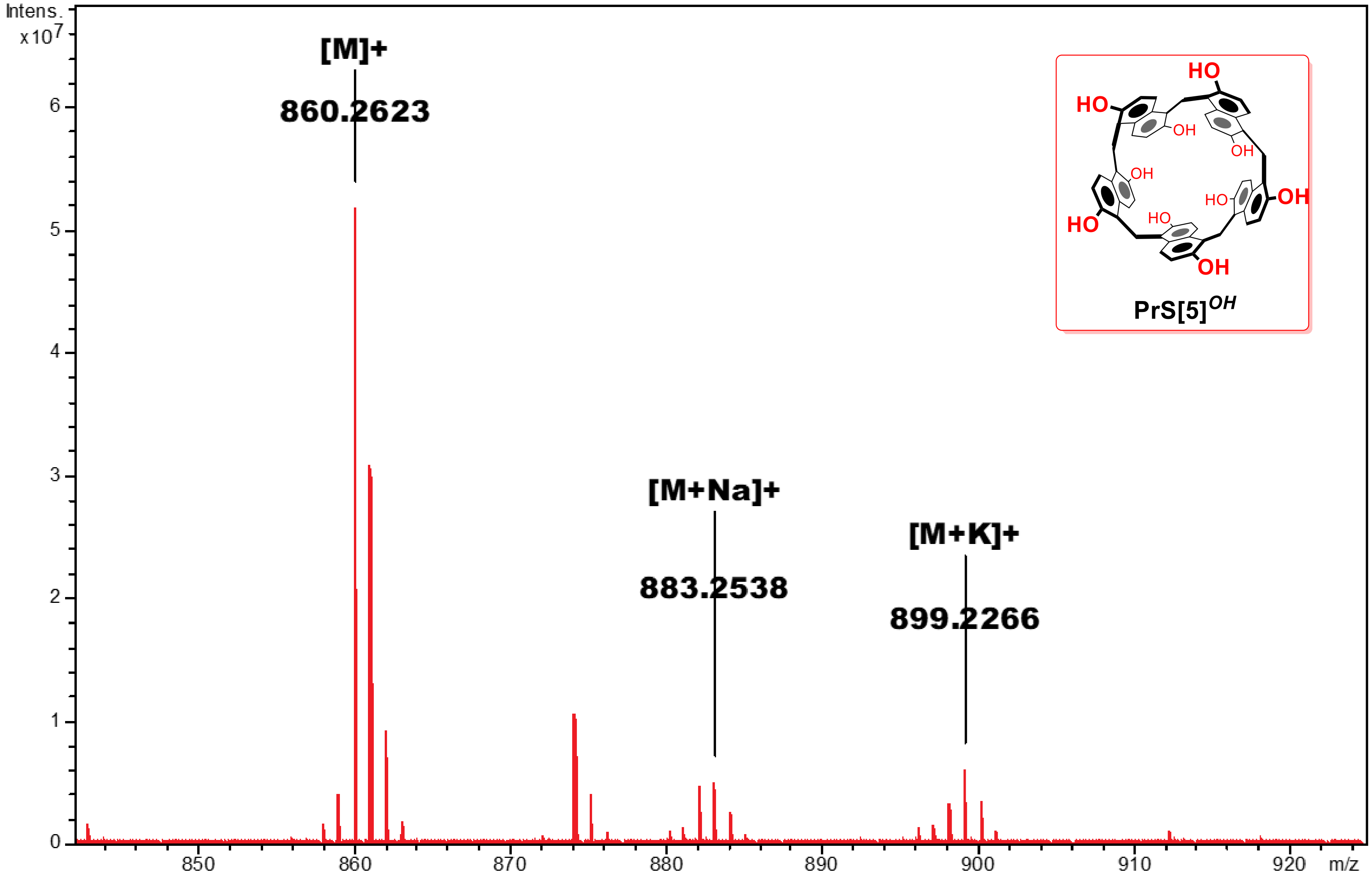

Figure S10: Significant portion of the HR MALDI FT-ICR mass spectrum of **PrS[5]^{OH}** [M]$^{+}$, [M+Na]$^{+}$ and [M+K]$^{+}$.

Copies of 1D NMR and HR mass spectrum of PrS[6]^{OH}

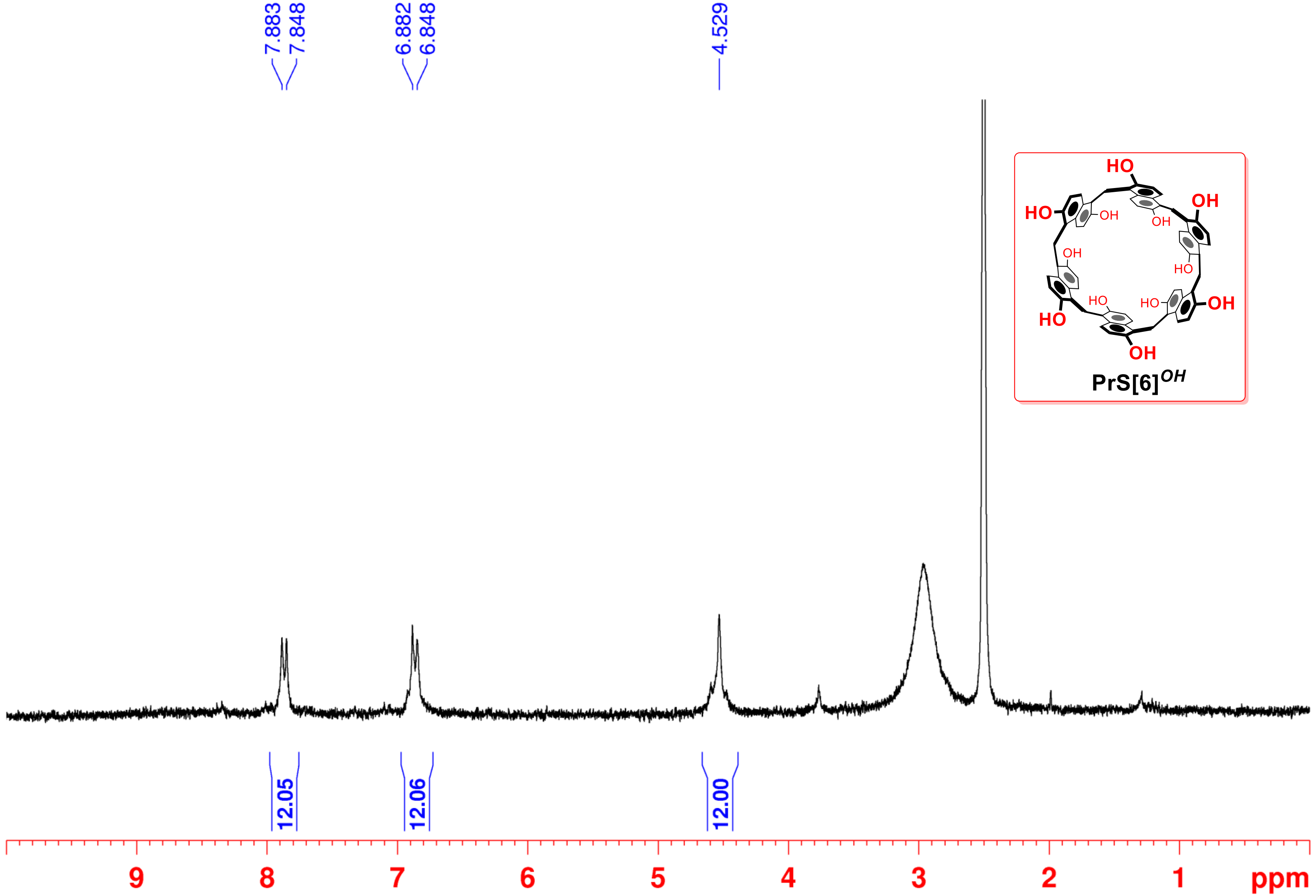

Figure S11: ^{1}H NMR spectrum of **PrS[6]^{OH}** (DMSO-d_6, 300 MHz, 373 K).

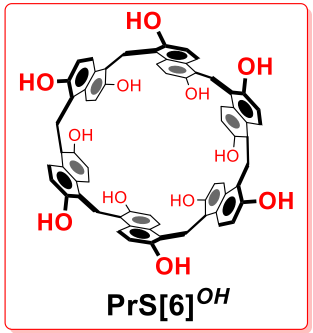

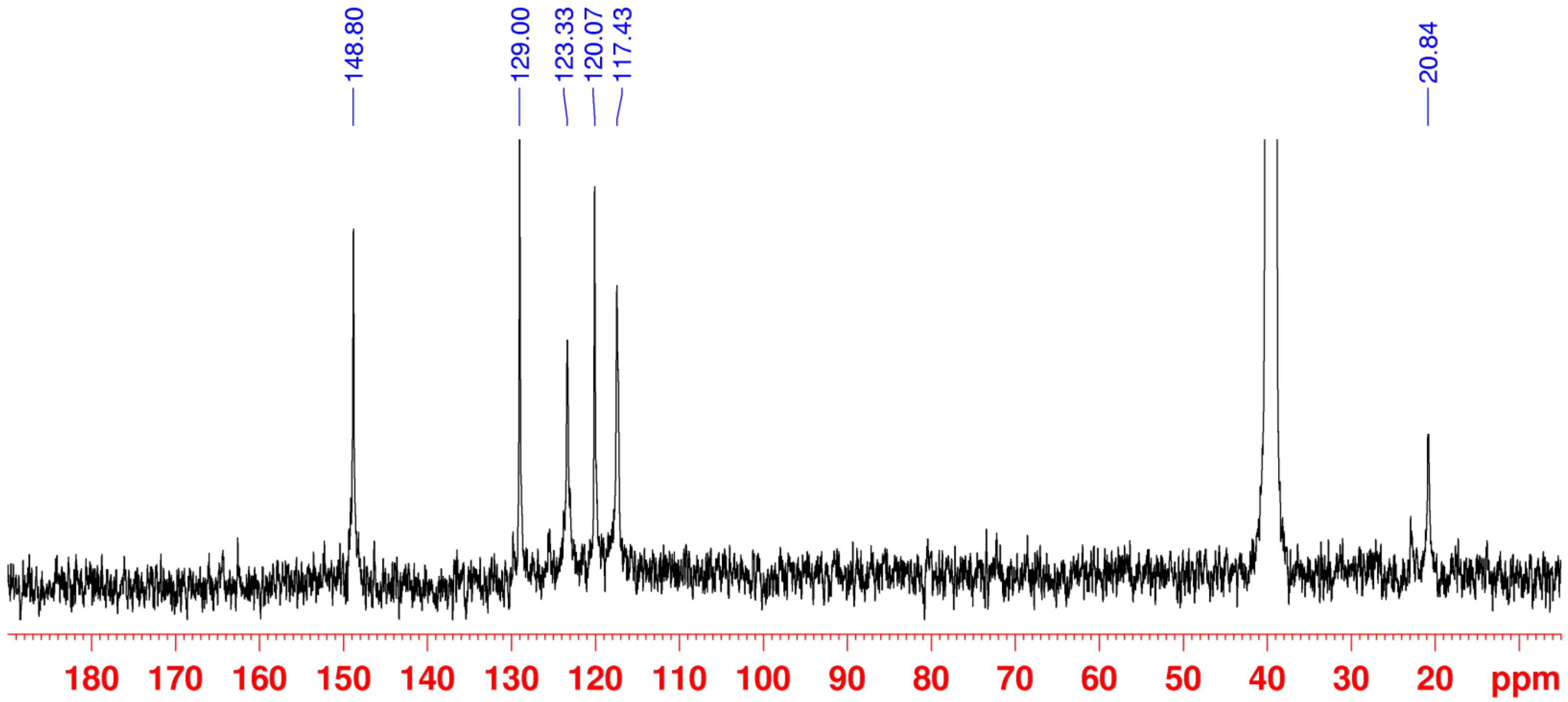

Figure S12: ^{13}C NMR spectrum of **PrS[6]^{OH}** (DMSO-d_6, 100 MHz, 298 K).

HT NMR studies of PrS[6]^{OH}

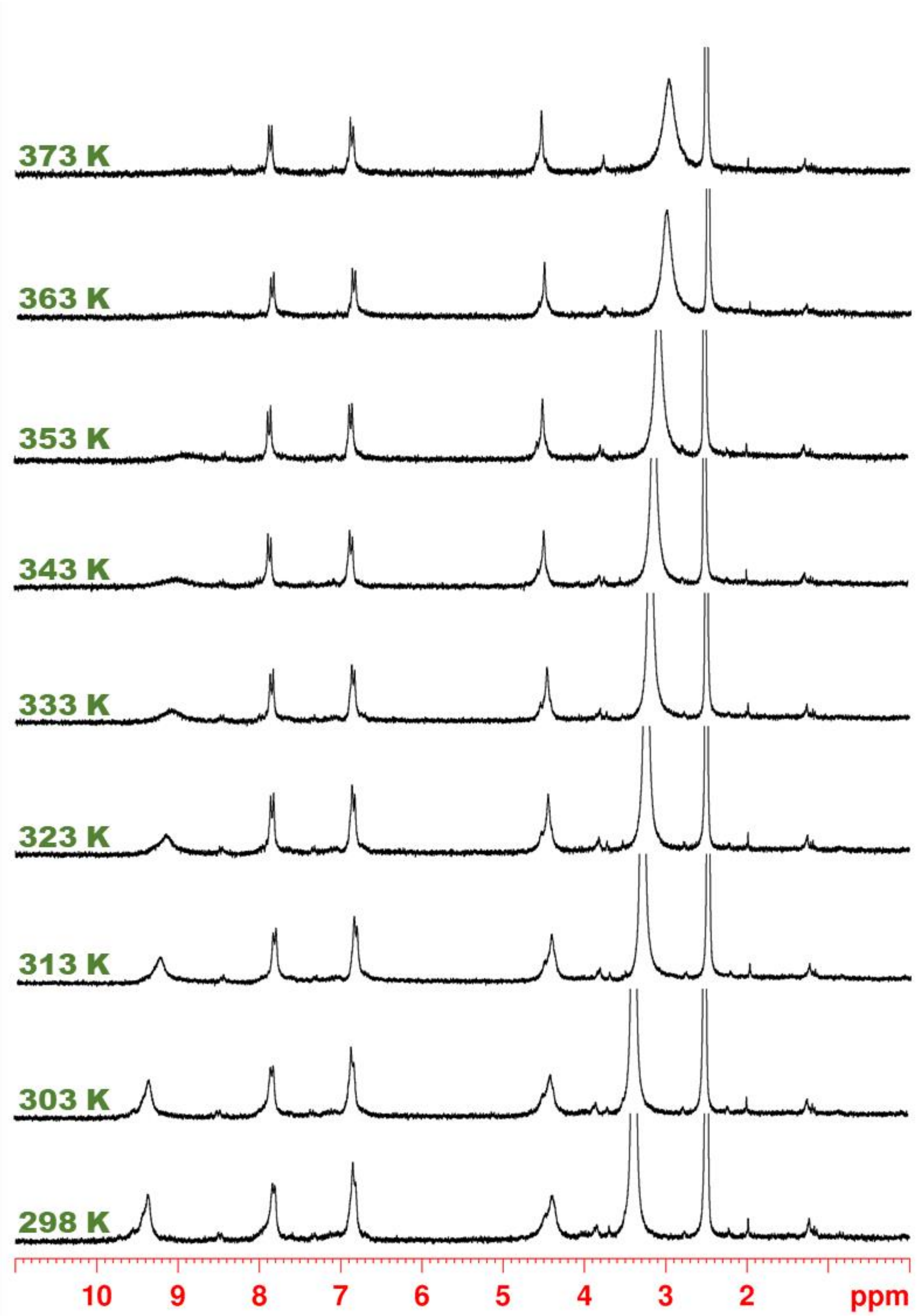

Figure S13: ^{1}H NMR spectra of **PrS[6]^{OH}** (250 MHz, DMSO-d_6) at (from bottom to top): 298, 303, 313, 323, 333, 343, 353, 363 and 373 K.

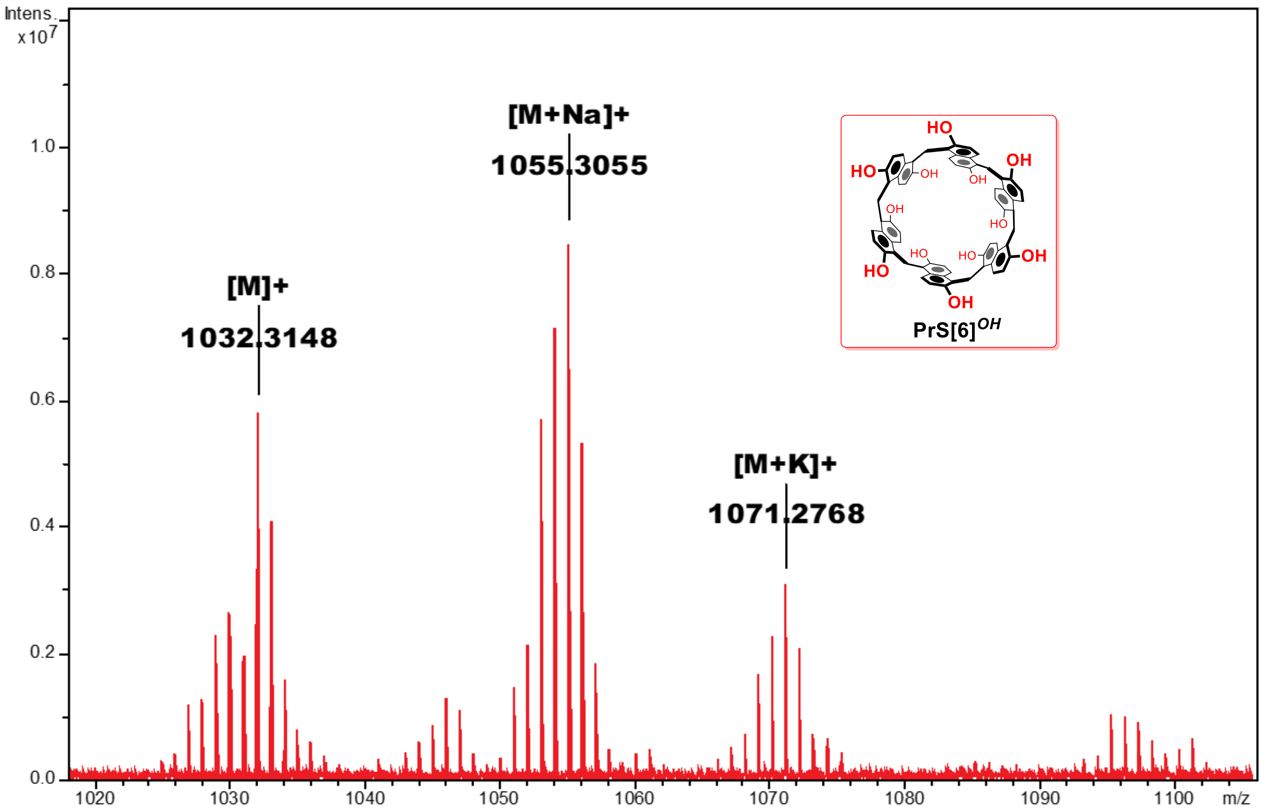

Figure S14: Significant portion of the HR MALDI FT-ICR mass spectrum of **PrS[6]**^{OH} $[M]^+$, $[M+Na]^+$ and $[M+K]^+$.

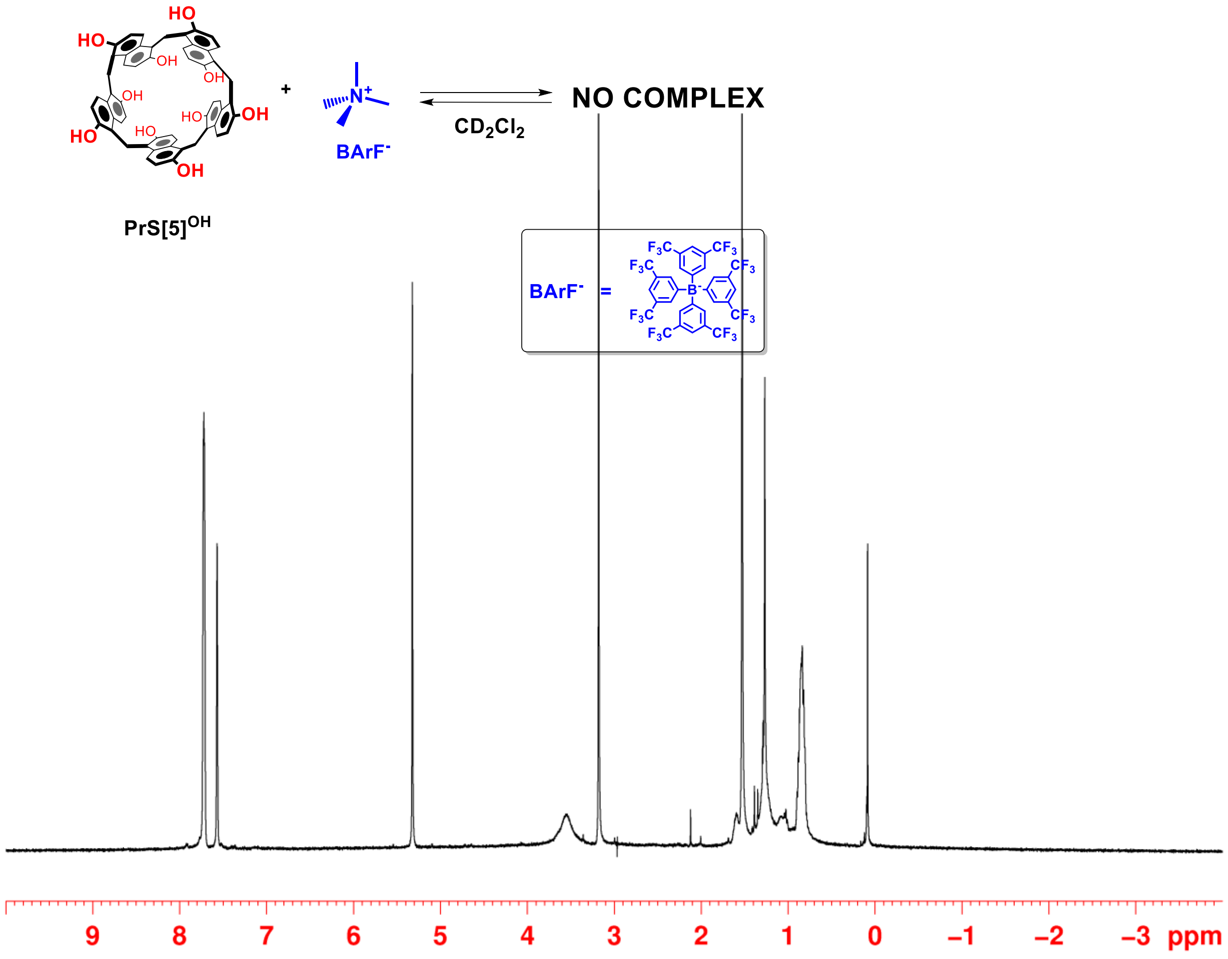

Figure S15: ^{1}H NMR spectrum (400 MHz, CD_2Cl_2, 298 K) of **PrS[5]*OH*** in presence of 1 equivalent of tetramethylammonium (as Barfate salt).

Conversion of PrS[5]^{Me} to PrS[6]^{Me} in the Presence of TFA

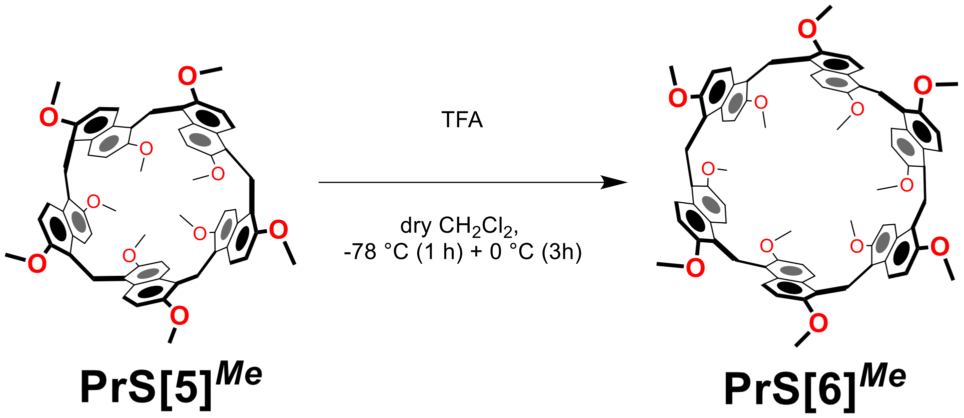

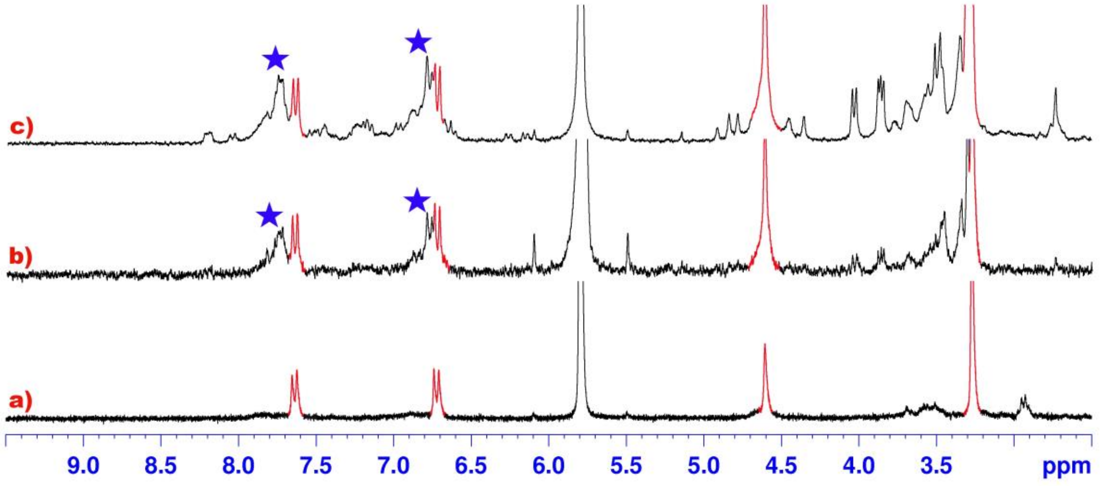

Figure S16. ^{1}H NMR spectra (393 K, 300 MHz, TCDE) of: a) **PrS[6]^{Me}**; b) crude product of the reaction after 2 h at 273 K ; c) crude product of the reaction after 3 h at 273 K. In red, ^{1}H NMR signals of **PrS[6]^{Me}**, marked with blue star the ^{1}H NMR signals of **PrS[5]^{Me}**.

Conversion of PrS[5]^{Me} to *c*-PrS[5]^{Me} in the Presence of

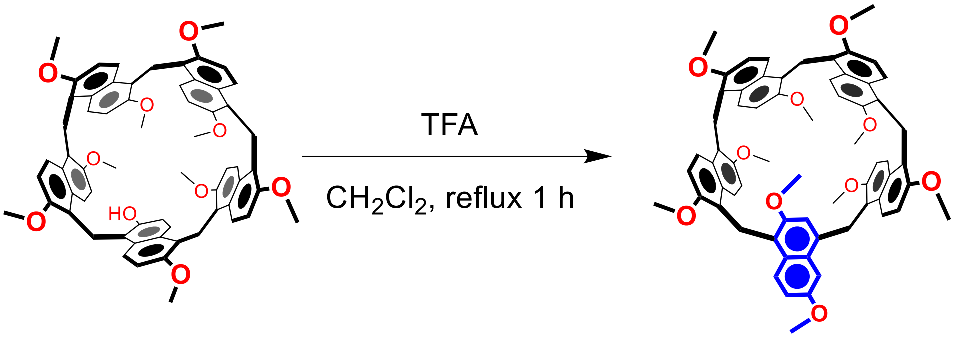

PrS[5]^{Me} **c-PrS[5]^{Me}**

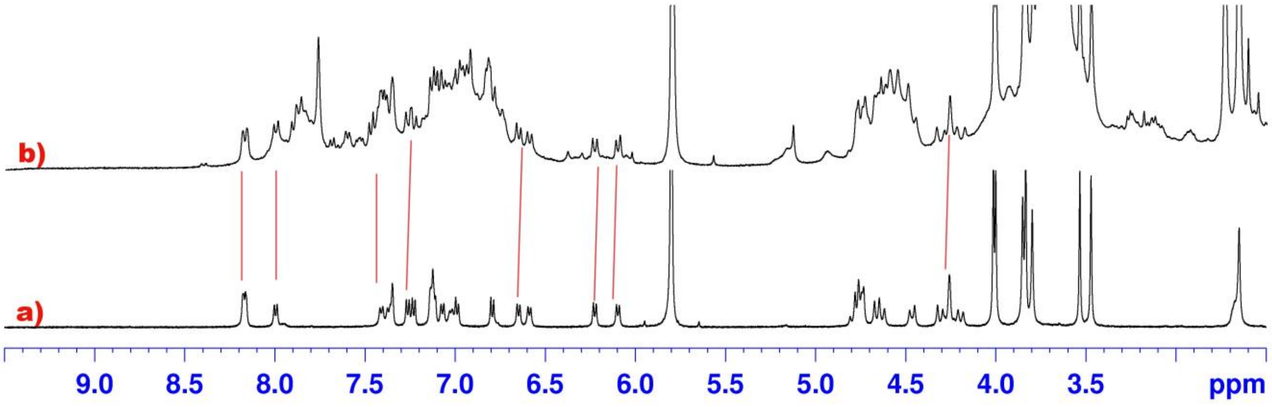

Figure S17. ^{1}H NMR spectra (298 K, 400 MHz, TCDE) of: a) *c*-**PrS[5]^{Me}**; b) crude product of the reaction after 1 h at reflux of CH_2Cl_2.

Reaction of PrS[6]^{Me} in the Presence of TFA

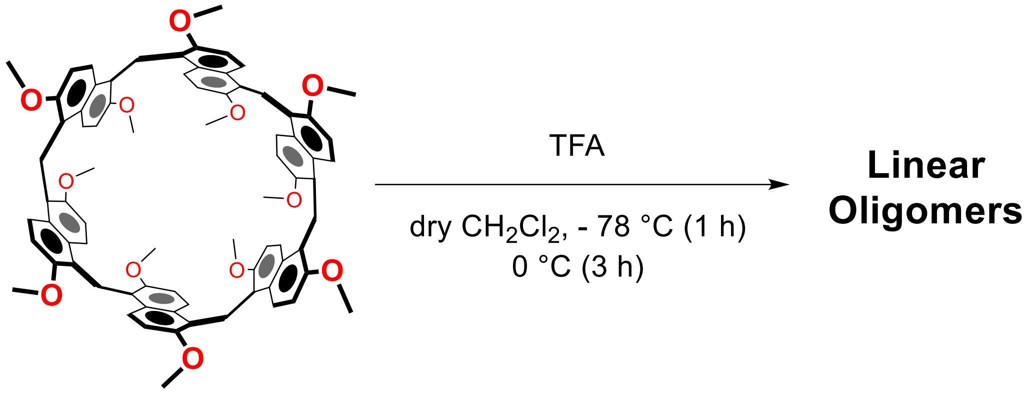

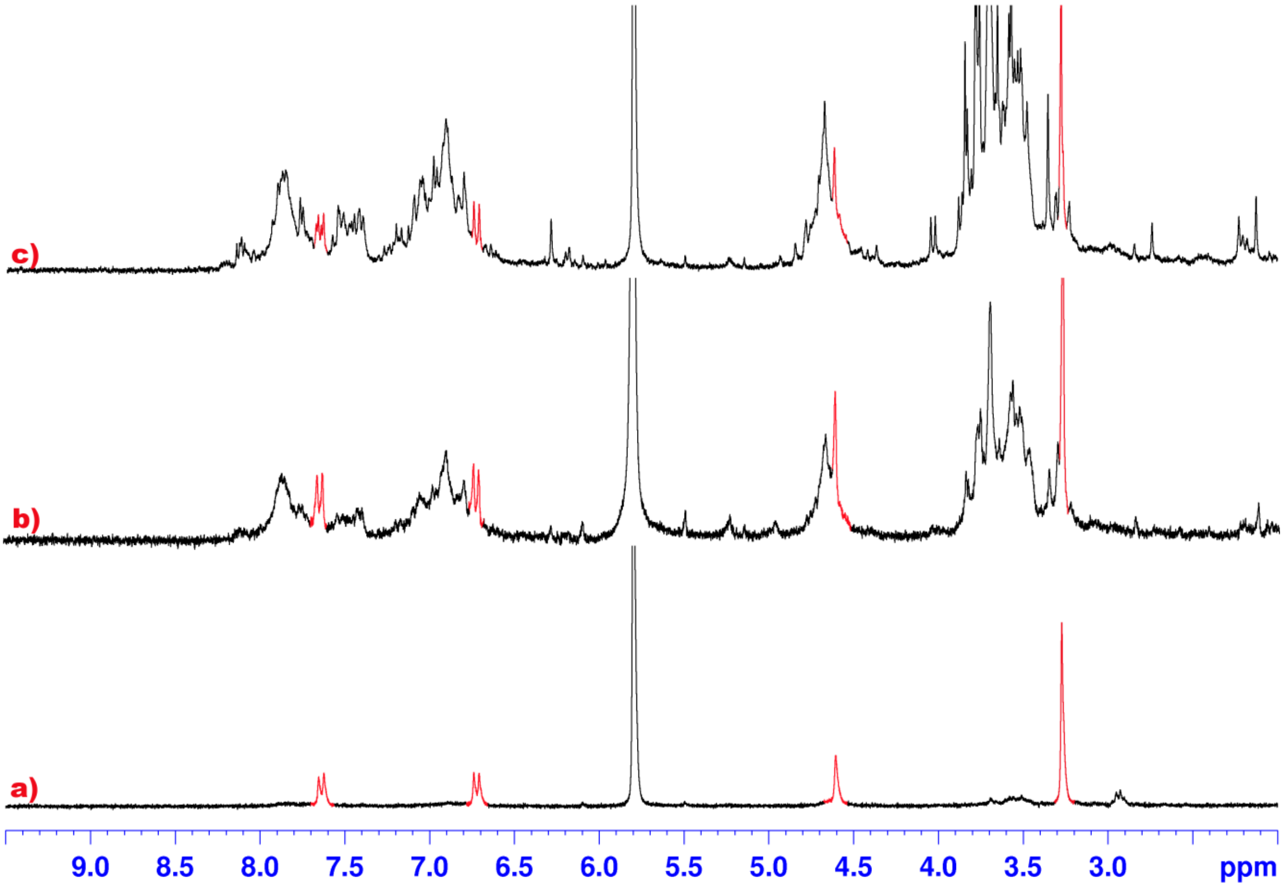

Figure S18. ^{1}H NMR spectra (393 K, 300 MHz, TCDE) of: a) **PrS[6]^{Me}**; b) crude product of the reaction after 2 h at 273 K ; c) crude product of the reaction after 3 h at 273 K. In red, ^{1}H NMR signals of **PrS[6]^{Me}**.

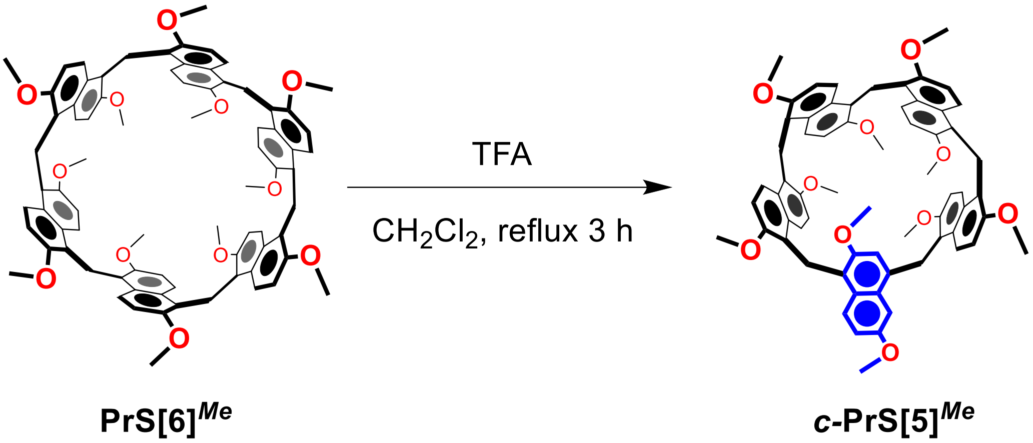

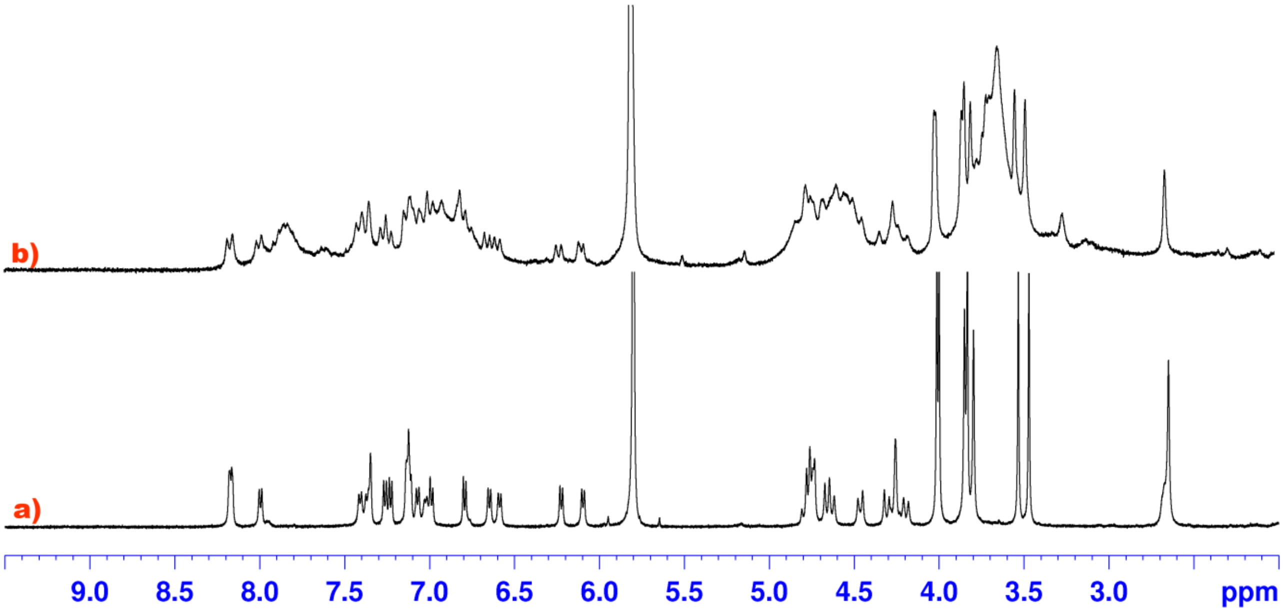

Figure S19. ^{1}H NMR spectra (298 K, 400 MHz, TCDE) of: a) *c*-**PrS[5]**^{Me}; b) crude product of the reaction after 3 h at reflux of CH_2Cl_2.

Computational details

Conformational studies have been performed using CREST software[5] which makes use of the xTB program.[6] All calculations executed with xTB were performed using the 6.3.2 release version of the program at GFN2-xTB level of theory, whereas the CREST version was the 2.10.2 with all parameters set to the default values.

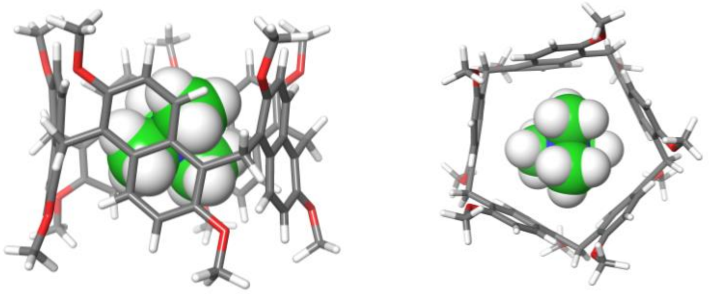

Figure S20. Optimzed structure of **1**$^{+}$**@PrS[5]**^{Me} complex

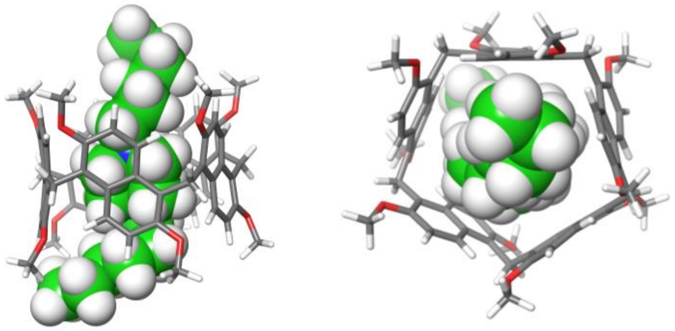

Figure S21. Optimized structure of **3**$^{2+}$**@PrS[5]**^{Me} complex

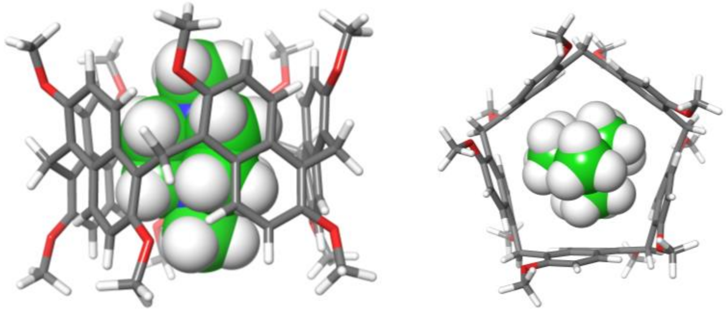

Figure S22. Optimized structure of **2**$^{+}$**@PrS[5]**^{Me} complex

Coordinates of 1^{+}@PrS[5]^{Me} ; 2^{2+}@PrS[5]^{Me}; 3^{2+}@PrS[5]^{Me} complexes

2^{2+}@PrS[5]^{Me}

C	2.25200000	-2.67100000	2.91500000
C	0.57700000	-4.39900000	1.46200000
C	0.34500000	-4.15000000	2.81000000
C	1.16200000	-3.24100000	3.50800000
C	2.62400000	-3.00000000	1.59400000
C	1.74000000	-3.84700000	0.85600000
C	2.12800000	-4.20400000	-0.45300000
C	3.35400000	-3.86000000	-0.94700000
C	4.26200000	-3.10300000	-0.18300000
C	3.87800000	-2.60100000	1.05500000
C	4.85700000	-1.77200000	1.85600000
C	-0.32100000	-5.36400000	0.71900000
O	5.52300000	-2.82700000	-0.61900000
O	-0.69100000	-4.80200000	3.40800000
C	6.06900000	-3.57500000	-1.68400000
C	-0.76300000	-4.84600000	4.81700000
C	-2.70800000	-3.52700000	-2.37300000
C	-1.18600000	-4.73000000	-0.34700000
C	-0.96400000	-5.04200000	-1.68100000
C	-1.72700000	-4.42200000	-2.68600000
C	-2.29000000	-3.90000000	-0.01300000
C	-3.04900000	-3.25300000	-1.03200000
C	-4.16800000	-2.44300000	-0.70200000
C	-4.61700000	-2.42500000	0.61100000
C	-3.88000000	-3.07800000	1.61500000
C	-2.73300000	-3.75700000	1.31800000
O	0.02700000	-5.93900000	-1.97100000
O	-5.76800000	-1.74100000	0.88100000
C	-4.92600000	-1.67600000	-1.76200000
C	-2.69800000	1.61300000	-3.46100000
C	-2.97800000	0.49800000	-4.20000000
C	-3.74000000	-0.55600000	-3.66600000
C	-4.14900000	-0.51300000	-2.33900000
C	-3.20900000	1.77300000	-2.15600000
C	-3.92600000	0.67400000	-1.59200000
C	-4.49900000	0.85900000	-0.31500000
C	-4.46000000	2.07200000	0.31000000
C	-3.79500000	3.16700000	-0.27000000
C	-3.10100000	3.00900000	-1.46200000
O	-4.09300000	-1.64600000	-4.40700000
O	-3.78800000	4.40200000	0.31400000
C	-2.37300000	4.19400000	-2.05700000
C	1.93500000	3.88900000	-2.22700000
C	-0.86400000	4.07900000	-2.09100000
C	-0.21600000	3.99600000	-3.31600000
C	1.18300000	3.86500000	-3.36600000
C	-0.08100000	4.18900000	-0.91100000
C	1.34400000	4.10500000	-0.96400000
C	2.13400000	4.34500000	0.19200000
C	1.50500000	4.76000000	1.35900000
C	0.10100000	4.78200000	1.43000000
C	-0.66700000	4.47800000	0.34100000
O	-0.98200000	4.02500000	-4.44600000
O	2.29100000	5.12500000	2.41200000
C	3.64500000	4.27600000	0.14400000
C	5.09700000	0.23700000	-0.30400000
C	5.28800000	1.16100000	-1.29100000
C	4.84700000	2.48700000	-1.13800000
C	4.18100000	2.86700000	0.01900000
C	4.09300000	1.94400000	1.09400000
C	4.52800000	0.59400000	0.93500000
C	4.46900000	-0.31900000	2.02100000
C	4.14600000	0.16100000	3.28200000
C	3.73500000	1.49600000	3.44400000

C	3.67100000	2.34600000	2.37900000
O	5.03100000	3.43300000	-2.10700000
O	4.21700000	-0.70900000	4.33500000
C	-4.01500000	-1.57900000	-5.81500000
C	-6.44700000	-1.98300000	2.09500000
C	0.03400000	-6.57100000	-3.23300000
C	-4.75000000	4.70500000	1.30100000
C	-0.37200000	4.30700000	-5.68700000
C	4.28000000	-0.19600000	5.64900000
C	5.99100000	3.20900000	-3.11700000
C	1.73000000	5.87900000	3.46500000
H	2.88300000	-2.03400000	3.50500000
H	0.95400000	-3.02300000	4.54300000
H	1.49300000	-4.83400000	-1.04600000
H	3.64400000	-4.21500000	-1.92300000
H	4.96300000	-2.20800000	2.85000000
H	5.82800000	-1.83400000	1.36600000
H	-0.95500000	-5.86200000	1.45200000
H	0.29400000	-6.12800000	0.24400000
H	5.95200000	-4.64900000	-1.52100000
H	5.62400000	-3.29500000	-2.64500000
H	0.18600000	-5.16100000	5.25700000
H	-1.06300000	-3.88000000	5.23700000
H	-3.27900000	-3.08900000	-3.17000000
H	-1.54900000	-4.66700000	-3.72100000
H	-4.22900000	-3.05800000	2.63500000
H	-5.85300000	-1.31000000	-1.32100000
H	-5.19300000	-2.34600000	-2.57900000
H	-2.15500000	2.41500000	-3.92400000
H	-2.63500000	0.44600000	-5.22100000
H	-5.05000000	0.06000000	0.14400000
H	-4.97500000	2.19500000	1.24900000
H	-2.64900000	5.07800000	-1.48300000
H	-2.71800000	4.34600000	-3.08000000
H	3.00300000	3.82500000	-2.31400000
H	1.67800000	3.77500000	-4.32000000
H	-0.38400000	5.07300000	2.34800000
H	-1.73400000	4.55200000	0.42600000
H	4.03500000	4.73700000	1.05100000
H	4.00800000	4.85600000	-0.70300000
H	5.46100000	-0.76200000	-0.45700000
H	5.79800000	0.86800000	-2.19400000
H	3.48200000	1.86300000	4.42600000
H	3.37000000	3.36300000	2.54900000
H	-2.97900000	-1.61900000	-6.16600000
H	-4.50200000	-0.68000000	-6.20000000
H	-6.59000000	-3.05200000	2.26600000
H	-5.92300000	-1.53900000	2.94700000
H	-0.94800000	-6.97800000	-3.48200000
H	0.36500000	-5.89000000	-4.02400000
H	-5.75700000	4.44000000	0.97200000
H	-4.52700000	4.20800000	2.25000000
H	0.21300000	3.45700000	-6.05300000
H	0.26200000	5.19400000	-5.62800000
H	5.03600000	0.58700000	5.73600000
H	4.56200000	-1.03900000	6.27700000
H	6.95000000	2.90200000	-2.69500000
H	5.64900000	2.46200000	-3.84100000
H	1.16700000	6.73500000	3.08700000
H	1.08600000	5.26600000	4.10400000
H	-2.20800000	-4.25200000	2.11200000
H	2.57600000	6.23500000	4.05000000
H	3.31000000	0.18800000	5.98000000
H	7.12900000	-3.32800000	-1.69900000
H	-4.54700000	-2.45700000	-6.17700000
H	-1.19200000	4.49700000	-6.37800000
H	-4.69100000	5.78300000	1.44200000
H	-1.52800000	-5.58500000	5.04600000
H	-7.41800000	-1.50400000	1.98500000
H	6.10900000	4.16500000	-3.62200000
H	0.74900000	-7.38800000	-3.14500000

N	-0.20200000	0.12000000	0.98200000
C	-0.67200000	0.41000000	2.34400000
C	-0.79900000	1.06500000	0.01000000
C	-0.58600000	-1.25300000	0.58300000
C	1.27300000	0.23500000	0.90900000
C	1.75800000	-0.31300000	-0.43800000
N	0.61700000	-0.38600000	-1.37900000
C	1.09000000	-0.68000000	-2.74000000
C	-0.30800000	-1.44500000	-0.91300000
C	-0.09400000	0.91300000	-1.34200000
H	-0.30000000	-0.34700000	3.03200000
H	-1.76100000	0.41000000	2.36800000
H	-0.31100000	1.38700000	2.66100000
H	1.73100000	-0.33900000	1.72100000
H	1.55800000	1.28300000	1.03800000
H	2.53800000	0.33800000	-0.84200000
H	2.18900000	-1.31200000	-0.31700000
H	0.24000000	-0.81800000	-3.40500000
H	1.69000000	-1.58900000	-2.73400000
H	1.69700000	0.14500000	-3.10700000
H	-0.69100000	2.09900000	0.36100000
H	-1.86900000	0.85200000	-0.08000000
H	-0.83800000	0.96700000	-2.14800000
H	0.63100000	1.71800000	-1.50100000
H	-1.64700000	-1.42000000	0.78600000
H	-0.01600000	-1.97500000	1.17800000
H	0.14000000	-2.43000000	-1.07600000
H	-1.23100000	-1.39700000	-1.49800000

1 4 1.5 5 1.5 76 1.0
2 3 1.5 6 1.5 12 1.0
3 4 1.5 14 1.0
4 77 1.0
5 6 1.5 10 1.5
6 7 1.5
7 8 1.5 78 1.0
8 9 1.5 79 1.0
9 10 1.5 13 1.0
10 11 1.0
11 62 1.0 80 1.0 81 1.0
12 18 1.0 82 1.0 83 1.0
13 15 1.0
14 16 1.0
15 84 1.0 85 1.0 128 1.0
16 86 1.0 87 1.0 132 1.0
17 20 1.5 22 1.5 88 1.0
18 19 1.5 21 1.5
19 20 1.5 27 1.0
20 89 1.0
21 22 1.5 26 1.5
22 23 1.5
23 24 1.5 29 1.0
24 25 1.5 28 1.0
25 26 1.5 90 1.0
26 125 1.0
27 70 1.0
28 69 1.0
29 33 1.0 91 1.0 92 1.0
30 31 1.5 34 1.5 93 1.0
31 32 1.5 94 1.0
32 33 1.5 40 1.0
33 35 1.5
34 35 1.5 39 1.5
35 36 1.5
36 37 1.5 95 1.0
37 38 1.5 96 1.0
38 39 1.5 41 1.0
39 42 1.0
40 68 1.0
41 71 1.0
42 44 1.0 97 1.0 98 1.0

43 46 1.5 48 1.5 99 1.0
44 45 1.5 47 1.5
45 46 1.5 53 1.0
46 100 1.0
47 48 1.5 52 1.5
48 49 1.5
49 50 1.5 55 1.0
50 51 1.5 54 1.0
51 52 1.5 101 1.0
52 102 1.0
53 72 1.0
54 75 1.0
55 59 1.0 103 1.0 104 1.0
56 57 1.5 61 1.5 105 1.0
57 58 1.5 106 1.0
58 59 1.5 66 1.0
59 60 1.5
60 61 1.5 65 1.5
61 62 1.5
62 63 1.5
63 64 1.5 67 1.0
64 65 1.5 107 1.0
65 108 1.0
66 74 1.0
67 73 1.0
68 109 1.0 110 1.0 129 1.0
69 111 1.0 112 1.0 133 1.0
70 113 1.0 114 1.0 135 1.0
71 115 1.0 116 1.0 131 1.0
72 117 1.0 118 1.0 130 1.0
73 119 1.0 120 1.0 127 1.0
74 121 1.0 122 1.0 134 1.0
75 123 1.0 124 1.0 126 1.0
76
77
78
79
80
81
82
83
84
85
86
87
88
89
90
91
92
93
94
95
96
97
98
99
100
101
102
103
104
105
106
107
108
109
110
111
112
113

114
115
116
117
118
119
120
121
122
123
124
125
126
127
128
129
130
131
132
133
134
135
136 137 1.0 138 1.0 139 1.0 140 1.0
137 146 1.0 147 1.0 148 1.0
138 145 1.0 156 1.0 157 1.0
139 144 1.0 160 1.0 161 1.0
140 141 1.0 149 1.0 150 1.0
141 142 1.0 151 1.0 152 1.0
142 143 1.0 144 1.0 145 1.0
143 153 1.0 154 1.0 155 1.0
144 162 1.0 163 1.0
145 158 1.0 159 1.0
146
147
148
149
150
151
152
153
154
155
156
157
158
159
160
161
162
163

3^{2+}@PrS[5]^{Me}

C	2.57900000	-2.80200000	3.11800000
C	0.94000000	-4.51900000	1.61400000
C	0.64700000	-4.23700000	2.94200000
C	1.45900000	-3.35000000	3.67200000
C	3.01000000	-3.17100000	1.82600000
C	2.17300000	-4.05500000	1.07900000
C	2.66300000	-4.51500000	-0.16100000
C	3.91600000	-4.18900000	-0.59500000
C	4.74200000	-3.32800000	0.14800000
C	4.27200000	-2.75700000	1.32300000
C	5.16000000	-1.80800000	2.09800000
C	0.00300000	-5.40200000	0.81700000
O	6.00800000	-3.00800000	-0.25400000
O	-0.45300000	-4.82900000	3.49500000
C	6.65100000	-3.81400000	-1.21700000

C	-0.58500000	-4.86400000	4.90000000
C	-2.29300000	-3.51700000	-2.33700000
C	-0.79100000	-4.70300000	-0.26900000
C	-0.51400000	-4.97900000	-1.60100000
C	-1.25000000	-4.35000000	-2.62400000
C	-1.91600000	-3.88300000	0.03400000
C	-2.70000000	-3.29000000	-1.00500000
C	-3.90600000	-2.60000000	-0.70800000
C	-4.35000000	-2.55500000	0.60800000
C	-3.54800000	-3.08000000	1.63600000
C	-2.36600000	-3.70700000	1.36000000
O	0.48600000	-5.86700000	-1.87600000
O	-5.56500000	-1.98600000	0.84800000
C	-4.78100000	-2.02900000	-1.80200000
C	-2.93100000	1.30700000	-3.80900000
C	-3.26100000	0.17500000	-4.49700000
C	-3.88500000	-0.90800000	-3.85400000
C	-4.18600000	-0.83000000	-2.50300000
C	-3.31600000	1.48600000	-2.46500000
C	-4.00100000	0.40600000	-1.83100000
C	-4.55200000	0.63600000	-0.55400000
C	-4.49200000	1.86900000	0.02800000
C	-3.79700000	2.92600000	-0.58500000
C	-3.12700000	2.72100000	-1.78400000
O	-4.18200000	-2.07200000	-4.50800000
O	-3.74100000	4.17400000	-0.02600000
C	-2.32100000	3.85200000	-2.38800000
C	1.98900000	3.44900000	-2.42600000
C	-0.81700000	3.66500000	-2.36700000
C	-0.14200000	3.45800000	-3.56400000
C	1.25700000	3.30900000	-3.57000000
C	-0.05400000	3.85500000	-1.18000000
C	1.37300000	3.77400000	-1.19700000
C	2.14100000	4.09900000	-0.04600000
C	1.48800000	4.59400000	1.07500000
C	0.08300000	4.62200000	1.11300000
C	-0.66200000	4.23900000	0.03500000
O	-0.87200000	3.41100000	-4.71700000
O	2.25000000	5.02600000	2.11900000
C	3.65400000	4.03900000	-0.05600000
C	5.26600000	0.03300000	-0.20400000
C	5.42000000	0.89200000	-1.25400000
C	4.92700000	2.20700000	-1.19400000
C	4.23200000	2.64100000	-0.07400000
C	4.16700000	1.79000000	1.06300000
C	4.67200000	0.45500000	1.00500000
C	4.65700000	-0.38200000	2.15300000
C	4.27000000	0.16300000	3.37000000
C	3.76900000	1.47500000	3.42700000
C	3.68400000	2.24800000	2.30500000
O	5.08300000	3.08600000	-2.23100000
O	4.38000000	-0.62300000	4.48200000
C	-4.38200000	-2.03500000	-5.90600000
C	-6.19100000	-2.18900000	2.09600000
C	0.56500000	-6.44500000	-3.16200000
C	-4.69000000	4.53200000	0.95600000
C	-0.22200000	3.64700000	-5.94900000
C	4.36500000	-0.01800000	5.75700000
C	6.09500000	2.84800000	-3.18600000
C	1.66700000	5.85800000	3.10100000
H	3.18100000	-2.14400000	3.71600000
H	1.20600000	-3.11100000	4.69200000
H	2.07100000	-5.18900000	-0.75100000
H	4.28200000	-4.62100000	-1.51300000
H	5.26500000	-2.16800000	3.12100000
H	6.15000000	-1.82300000	1.64200000
H	-0.69200000	-5.86600000	1.51700000
H	0.57400000	-6.20100000	0.34600000
H	6.59200000	-4.87300000	-0.95700000
H	6.23700000	-3.65500000	-2.21900000
H	0.32900000	-5.22000000	5.37900000

H	-0.85700000	-3.88300000	5.30400000
H	-2.85800000	-3.09600000	-3.14800000
H	-1.01600000	-4.56100000	-3.65500000
H	-3.88400000	-3.02500000	2.65900000
H	-5.73900000	-1.75700000	-1.36100000
H	-4.96800000	-2.80000000	-2.54900000
H	-2.43400000	2.10300000	-4.33300000
H	-3.03800000	0.11200000	-5.54900000
H	-5.10300000	-0.14300000	-0.06100000
H	-5.00100000	2.03400000	0.96400000
H	-2.56900000	4.76500000	-1.84500000
H	-2.62200000	3.99500000	-3.42500000
H	3.06000000	3.39300000	-2.48900000
H	1.77000000	3.13000000	-4.50200000
H	-0.42100000	4.97500000	1.99800000
H	-1.73100000	4.30800000	0.09800000
H	4.01400000	4.56700000	0.82700000
H	4.02900000	4.56900000	-0.93100000
H	5.67900000	-0.95500000	-0.28000000
H	5.95100000	0.55900000	-2.13200000
H	3.46300000	1.88700000	4.37400000
H	3.31100000	3.25100000	2.39500000
H	-3.43400000	-1.98700000	-6.45200000
H	-5.01500000	-1.19300000	-6.19500000
H	-6.22900000	-3.24800000	2.35800000
H	-5.69500000	-1.62600000	2.89300000
H	-0.39900000	-6.84600000	-3.48200000
H	0.93300000	-5.72800000	-3.90400000
H	-5.70700000	4.29800000	0.63300000
H	-4.48500000	4.04500000	1.91500000
H	0.38200000	2.78800000	-6.26100000
H	0.40300000	4.54100000	-5.90600000
H	4.68800000	-0.79400000	6.45000000
H	3.36100000	0.31500000	6.03800000
H	7.04900000	2.61600000	-2.70800000
H	5.82000000	2.04100000	-3.87400000
H	1.13500000	6.69700000	2.64700000
H	0.99100000	5.30100000	3.75600000
H	-1.81200000	-4.13700000	2.17300000
H	2.50000000	6.23800000	3.69000000
H	5.06000000	0.82300000	5.80900000
H	7.69300000	-3.50000000	-1.21000000
H	-4.88500000	-2.96700000	-6.15500000
H	-1.01900000	3.80100000	-6.67400000
H	-4.59000000	5.60900000	1.07600000
H	-1.39300000	-5.56600000	5.09800000
H	-7.20600000	-1.81300000	1.97500000
H	6.19000000	3.77600000	-3.74800000
H	1.28000000	-7.26000000	-3.07100000
N	-0.33500000	-0.19200000	0.53300000
C	0.38200000	-1.48700000	0.53500000
C	0.67600000	0.88500000	0.52100000
C	-1.10400000	-0.10500000	-0.73000000
C	-0.14900000	0.05600000	-1.91900000
N	1.19800000	-0.40900000	-1.52100000
C	2.16000000	-0.47200000	-2.66800000
C	1.73000000	0.56700000	-0.54200000
C	1.04900000	-1.70700000	-0.82700000
C	2.14800000	-1.73000000	-3.53600000
C	0.94200000	-1.94600000	-4.45400000
C	0.68500000	-0.79500000	-5.42700000
C	-0.32200000	-1.20400000	-6.50100000
C	-0.65800000	-0.05100000	-7.43700000
C	-1.28400000	-0.08600000	1.68200000
C	-0.64500000	-0.08600000	3.07400000
C	-0.44800000	1.31200000	3.67000000
C	0.21400000	1.22900000	5.04600000
C	0.61800000	2.59800000	5.59300000
C	-0.56900000	3.51400000	5.86500000
H	-1.77900000	0.75400000	-0.69500000
H	-1.71900000	-1.00700000	-0.82700000

H	-0.51500000	-0.52700000	-2.76300000
H	-0.08400000	1.10400000	-2.23100000
H	-0.32600000	-2.30000000	0.73200000
H	1.12800000	-1.49100000	1.33200000
H	2.03100000	-2.16400000	-0.67900000
H	0.44900000	-2.38000000	-1.44500000
H	1.16500000	0.95200000	1.49300000
H	0.17600000	1.83900000	0.31800000
H	2.01100000	1.49100000	-1.06000000
H	2.63300000	0.14900000	-0.08700000
H	3.16000000	-0.38200000	-2.23300000
H	1.97800000	0.41100000	-3.28400000
H	3.02900000	-1.63600000	-4.17500000
H	2.30300000	-2.61800000	-2.92200000
H	1.15700000	-2.83900000	-5.04600000
H	0.03800000	-2.16600000	-3.88200000
H	0.29700000	0.08200000	-4.90100000
H	1.62300000	-0.51300000	-5.90900000
H	0.08700000	-2.03200000	-7.08200000
H	-1.23000000	-1.56200000	-6.01100000
H	-1.36800000	-0.37000000	-8.19500000
H	0.23700000	0.30200000	-7.94400000
H	-1.09400000	0.78100000	-6.88700000
H	-1.97500000	-0.92800000	1.58300000
H	-1.85400000	0.83600000	1.54800000
H	-1.31100000	-0.63600000	3.74000000
H	0.30100000	-0.62800000	3.07600000
H	0.17100000	1.93800000	3.02400000
H	-1.42100000	1.79500000	3.76400000
H	-0.46600000	0.74700000	5.75000000
H	1.10600000	0.60200000	4.96500000
H	1.16300000	2.45000000	6.52600000
H	1.29900000	3.08000000	4.88600000
H	-0.23300000	4.43000000	6.34200000
H	-1.27800000	3.02900000	6.53200000
H	-1.08700000	3.78000000	4.94700000

1 4 1.5 5 1.5 76 1.0
2 3 1.5 6 1.5 12 1.0
3 4 1.5 14 1.0
4 77 1.0
5 6 1.5 10 1.5
6 7 1.5
7 8 1.5 78 1.0
8 9 1.5 79 1.0
9 10 1.5 13 1.0
10 11 1.0
11 62 1.0 80 1.0 81 1.0
12 18 1.0 82 1.0 83 1.0
13 15 1.0
14 16 1.0
15 84 1.0 85 1.0 128 1.0
16 86 1.0 87 1.0 132 1.0
17 20 1.5 22 1.5 88 1.0
18 19 1.5 21 1.5
19 20 1.5 27 1.0
20 89 1.0
21 22 1.5 26 1.5
22 23 1.5
23 24 1.5 29 1.0
24 25 1.5 28 1.0
25 26 1.5 90 1.0
26 125 1.0
27 70 1.0
28 69 1.0
29 33 1.0 91 1.0 92 1.0
30 31 1.5 34 1.5 93 1.0
31 32 1.5 94 1.0
32 33 1.5 40 1.0
33 35 1.5
34 35 1.5 39 1.5

35 36 1.5
36 37 1.5 95 1.0
37 38 1.5 96 1.0
38 39 1.5 41 1.0
39 42 1.0
40 68 1.0
41 71 1.0
42 44 1.0 97 1.0 98 1.0
43 46 1.5 48 1.5 99 1.0
44 45 1.5 47 1.5
45 46 1.5 53 1.0
46 100 1.0
47 48 1.5 52 1.5
48 49 1.5
49 50 1.5 55 1.0
50 51 1.5 54 1.0
51 52 1.5 101 1.0
52 102 1.0
53 72 1.0
54 75 1.0
55 59 1.0 103 1.0 104 1.0
56 57 1.5 61 1.5 105 1.0
57 58 1.5 106 1.0
58 59 1.5 66 1.0
59 60 1.5
60 61 1.5 65 1.5
61 62 1.5
62 63 1.5
63 64 1.5 67 1.0
64 65 1.5 107 1.0
65 108 1.0
66 74 1.0
67 73 1.0
68 109 1.0 110 1.0 129 1.0
69 111 1.0 112 1.0 133 1.0
70 113 1.0 114 1.0 135 1.0
71 115 1.0 116 1.0 131 1.0
72 117 1.0 118 1.0 130 1.0
73 119 1.0 120 1.0 127 1.0
74 121 1.0 122 1.0 134 1.0
75 123 1.0 124 1.0 126 1.0
76
77
78
79
80
81
82
83
84
85
86
87
88
89
90
91
92
93
94
95
96
97
98
99
100
101
102
103
104
105

106
107
108
109
110
111
112
113
114
115
116
117
118
119
120
121
122
123
124
125
126
127
128
129
130
131
132
133
134
135
136 137 1.0 138 1.0 139 1.0 150 1.0
137 144 1.0 160 1.0 161 1.0
138 143 1.0 164 1.0 165 1.0
139 140 1.0 156 1.0 157 1.0
140 141 1.0 158 1.0 159 1.0
141 142 1.0 143 1.0 144 1.0
142 145 1.0 168 1.0 169 1.0
143 166 1.0 167 1.0
144 162 1.0 163 1.0
145 146 1.0 170 1.0 171 1.0
146 147 1.0 172 1.0 173 1.0
147 148 1.0 174 1.0 175 1.0
148 149 1.0 176 1.0 177 1.0
149 178 1.0 179 1.0 180 1.0
150 151 1.0 181 1.0 182 1.0
151 152 1.0 183 1.0 184 1.0
152 153 1.0 185 1.0 186 1.0
153 154 1.0 187 1.0 188 1.0
154 155 1.0 189 1.0 190 1.0
155 191 1.0 192 1.0 193 1.0
156
157
158
159
160
161
162
163
164
165
166
167
168
169
170
171
172
173
174
175
176

177
178
179
180
181
182
183
184
185
186
187
188
189
190
191
192
193

1^+@PrS[5]^{Me}

C	2.41000000	-2.78100000	3.01400000
C	0.73100000	-4.52500000	1.59200000
C	0.48900000	-4.23800000	2.92700000
C	1.31800000	-3.33600000	3.61600000
C	2.78600000	-3.14600000	1.70600000
C	1.92200000	-4.03200000	0.99500000
C	2.34700000	-4.47000000	-0.27500000
C	3.57000000	-4.12800000	-0.77300000
C	4.42800000	-3.27500000	-0.06100000
C	4.02000000	-2.72200000	1.14200000
C	4.95100000	-1.78400000	1.88100000
C	-0.22300000	-5.42900000	0.83700000
O	5.68100000	-2.95000000	-0.51700000
O	-0.57100000	-4.86300000	3.53300000
C	6.22700000	-3.67200000	-1.59500000
C	-0.70400000	-4.78800000	4.93200000
C	-2.44000000	-3.32800000	-2.22000000
C	-1.03500000	-4.70800000	-0.21900000
C	-0.78800000	-4.95800000	-1.56100000
C	-1.47300000	-4.23400000	-2.55100000
C	-2.11000000	-3.84600000	0.12500000
C	-2.83700000	-3.14100000	-0.88100000
C	-3.97100000	-2.35300000	-0.54600000
C	-4.43300000	-2.37300000	0.76100000
C	-3.70500000	-3.04300000	1.75700000
C	-2.56600000	-3.73000000	1.45300000
O	0.13100000	-5.92700000	-1.87100000
O	-5.60800000	-1.71700000	1.03000000
C	-4.75600000	-1.60000000	-1.59800000
C	-2.84500000	1.84800000	-3.36500000
C	-3.08900000	0.72600000	-4.10200000
C	-3.72200000	-0.39200000	-3.53200000
C	-4.06600000	-0.38900000	-2.18900000
C	-3.25500000	1.94700000	-2.02100000
C	-3.87500000	0.80200000	-1.43800000
C	-4.36400000	0.92800000	-0.12200000
C	-4.32400000	2.12200000	0.53900000
C	-3.74400000	3.25800000	-0.05000000
C	-3.14400000	3.16700000	-1.29700000
O	-4.02400000	-1.51200000	-4.26300000
O	-3.74800000	4.48200000	0.56900000
C	-2.48600000	4.39200000	-1.89800000
C	1.79800000	4.01700000	-2.29400000
C	-0.97900000	4.29500000	-2.01600000
C	-0.39700000	4.22000000	-3.27200000
C	0.99200000	4.04900000	-3.39500000
C	-0.13400000	4.37700000	-0.87600000
C	1.28000000	4.23400000	-1.00100000
C	2.13300000	4.40500000	0.12300000

C	1.58200000	4.85600000	1.31300000
C	0.18800000	4.96700000	1.44600000
C	-0.64400000	4.69800000	0.39800000
O	-1.22100000	4.31500000	-4.36200000
O	2.44100000	5.17900000	2.33000000
C	3.63200000	4.21200000	0.02200000
C	4.88900000	0.09600000	-0.39400000
C	5.00700000	0.97500000	-1.43200000
C	4.63200000	2.32200000	-1.28800000
C	4.07000000	2.76600000	-0.10000000
C	4.02100000	1.87400000	1.00700000
C	4.43800000	0.51600000	0.87400000
C	4.47700000	-0.35100000	1.99800000
C	4.19200000	0.16800000	3.25100000
C	3.75700000	1.49700000	3.38200000
C	3.65400000	2.31500000	2.29400000
O	4.80500000	3.23800000	-2.29400000
O	4.35100000	-0.65800000	4.33300000
C	-3.91100000	-1.47000000	-5.66500000
C	-6.23700000	-1.91700000	2.27300000
C	0.23600000	-6.37500000	-3.20100000
C	-4.56100000	4.67700000	1.70100000
C	-0.65600000	4.47300000	-5.64100000
C	4.31400000	-0.11000000	5.62900000
C	5.58200000	2.89000000	-3.41400000
C	1.94500000	5.84700000	3.46500000
H	3.02700000	-2.11000000	3.58000000
H	1.10200000	-3.08400000	4.64200000
H	1.73000000	-5.14000000	-0.84200000
H	3.88100000	-4.53700000	-1.72100000
H	5.11200000	-2.16500000	2.88900000
H	5.91200000	-1.79600000	1.36600000
H	-0.89300000	-5.89300000	1.56000000
H	0.33900000	-6.22400000	0.34900000
H	6.20500000	-4.74900000	-1.41100000
H	5.71300000	-3.44900000	-2.53600000
H	0.21100000	-5.10000000	5.44100000
H	-0.98400000	-3.78100000	5.26000000
H	-2.95000000	-2.80600000	-3.00700000
H	-1.24500000	-4.39900000	-3.59200000
H	-4.05300000	-3.03300000	2.77700000
H	-5.69700000	-1.28300000	-1.14700000
H	-4.99500000	-2.27900000	-2.41700000
H	-2.38600000	2.68900000	-3.84800000
H	-2.80700000	0.71300000	-5.14200000
H	-4.84000000	0.09200000	0.35200000
H	-4.76300000	2.19000000	1.52200000
H	-2.74900000	5.25100000	-1.28300000
H	-2.89100000	4.56300000	-2.89500000
H	2.85500000	3.89800000	-2.43300000
H	1.43800000	3.95300000	-4.37200000
H	-0.23900000	5.28300000	2.38500000
H	-1.70200000	4.81000000	0.53800000
H	4.08800000	4.65100000	0.90900000
H	4.01100000	4.75200000	-0.84500000
H	5.21200000	-0.91700000	-0.53700000
H	5.41400000	0.62700000	-2.36800000
H	3.51800000	1.89300000	4.35600000
H	3.34400000	3.33100000	2.44300000
H	-2.86600000	-1.41000000	-5.98700000
H	-4.47500000	-0.63500000	-6.09000000
H	-6.38700000	-2.98000000	2.48200000
H	-5.67300000	-1.45700000	3.09200000
H	-0.73400000	-6.68400000	-3.60000000
H	0.67600000	-5.61200000	-3.85300000
H	-5.59600000	4.38400000	1.50700000
H	-4.18100000	4.13300000	2.57200000
H	-0.14700000	3.56200000	-5.97400000
H	0.04000000	5.31600000	-5.67200000
H	4.60800000	-0.92100000	6.29500000
H	3.30700000	0.22700000	5.89900000

H	6.56800000	2.52100000	-3.12200000
H	5.08000000	2.14500000	-4.04100000
H	1.39600000	6.75300000	3.19100000
H	1.30400000	5.19700000	4.07000000
H	-2.05000000	-4.24400000	2.24100000
H	2.82300000	6.12200000	4.04700000
H	5.02000000	0.71800000	5.73600000
H	7.26300000	-3.34200000	-1.67000000
H	-4.33900000	-2.40700000	-6.01900000
H	-1.49400000	4.67500000	-6.30600000
H	-4.52300000	5.74600000	1.90500000
H	-1.50800000	-5.47900000	5.18400000
H	-7.20600000	-1.42800000	2.19000000
H	5.69900000	3.81200000	-3.98400000
H	0.90000000	-7.23700000	-3.16600000
N	0.22300000	0.22900000	-0.15200000
C	0.53900000	1.25900000	0.85100000
C	-1.02300000	-0.46200000	0.22300000
C	1.32300000	-0.74700000	-0.21400000
C	0.05100000	0.86800000	-1.46600000
H	-1.28300000	-1.21500000	-0.52000000
H	-1.84700000	0.24800000	0.28900000
H	-0.90100000	-0.96300000	1.18100000
H	1.47400000	1.76000000	0.59900000
H	0.64800000	0.80200000	1.83200000
H	-0.25200000	2.00700000	0.88300000
H	1.09100000	-1.53100000	-0.93200000
H	1.47800000	-1.21500000	0.75800000
H	2.25200000	-0.25600000	-0.50500000
H	-0.16300000	0.11400000	-2.22100000
H	0.95400000	1.41100000	-1.74100000
H	-0.78000000	1.57300000	-1.44100000

1 4 1.5 5 1.5 76 1.0
2 3 1.5 6 1.5 12 1.0
3 4 1.5 14 1.0
4 77 1.0
5 6 1.5 10 1.5
6 7 1.5
7 8 1.5 78 1.0
8 9 1.5 79 1.0
9 10 1.5 13 1.0
10 11 1.0
11 62 1.0 80 1.0 81 1.0
12 18 1.0 82 1.0 83 1.0
13 15 1.0
14 16 1.0
15 84 1.0 85 1.0 128 1.0
16 86 1.0 87 1.0 132 1.0
17 20 1.5 22 1.5 88 1.0
18 19 1.5 21 1.5
19 20 1.5 27 1.0
20 89 1.0
21 22 1.5 26 1.5
22 23 1.5
23 24 1.5 29 1.0
24 25 1.5 28 1.0
25 26 1.5 90 1.0
26 125 1.0
27 70 1.0
28 69 1.0
29 33 1.0 91 1.0 92 1.0
30 31 1.5 34 1.5 93 1.0
31 32 1.5 94 1.0
32 33 1.5 40 1.0
33 35 1.5
34 35 1.5 39 1.5
35 36 1.5
36 37 1.5 95 1.0
37 38 1.5 96 1.0
38 39 1.5 41 1.0

39 42 1.0
40 68 1.0
41 71 1.0
42 44 1.0 97 1.0 98 1.0
43 46 1.5 48 1.5 99 1.0
44 45 1.5 47 1.5
45 46 1.5 53 1.0
46 100 1.0
47 48 1.5 52 1.5
48 49 1.5
49 50 1.5 55 1.0
50 51 1.5 54 1.0
51 52 1.5 101 1.0
52 102 1.0
53 72 1.0
54 75 1.0
55 59 1.0 103 1.0 104 1.0
56 57 1.5 61 1.5 105 1.0
57 58 1.5 106 1.0
58 59 1.5 66 1.0
59 60 1.5
60 61 1.5 65 1.5
61 62 1.5
62 63 1.5
63 64 1.5 67 1.0
64 65 1.5 107 1.0
65 108 1.0
66 74 1.0
67 73 1.0
68 109 1.0 110 1.0 129 1.0
69 111 1.0 112 1.0 133 1.0
70 113 1.0 114 1.0 135 1.0
71 115 1.0 116 1.0 131 1.0
72 117 1.0 118 1.0 130 1.0
73 119 1.0 120 1.0 127 1.0
74 121 1.0 122 1.0 134 1.0
75 123 1.0 124 1.0 126 1.0
76
77
78
79
80
81
82
83
84
85
86
87
88
89
90
91
92
93
94
95
96
97
98
99
100
101
102
103
104
105
106
107
108
109

110
111
112
113
114
115
116
117
118
119
120
121
122
123
124
125
126
127
128
129
130
131
132
133
134
135
136 137 1.0 138 1.0 139 1.0 140 1.0
137 144 1.0 145 1.0 146 1.0
138 141 1.0 142 1.0 143 1.0
139 147 1.0 148 1.0 149 1.0
140 150 1.0 151 1.0 152 1.0
141
142
143
144
145
146
147
148
149
150
151
152

References:

1) Furniss, B. S.; Hannaford, A. J.; Smith, P. W. G.; Tatchell, A. R. Vogel's Textbook of Practical Organic Chemistry, 5th ed.; Pearson: Harlow, 1989 and references cited therein.

2) Fulmer, G. R.; Miller, A. J. M.; Sherden, N. H.; Gottlieb, H. E.; Nudelman, A.; Stoltz, B.M.; Bercaw, J.E.; Goldberg, K.I. Chemical Shifts of Trace Impurities: Common Laboratory Solvents, Organics, and Gases in Deuterated Solvents Relevant to the Organometallic Chemist. *Organometallics* **2010**, *29*, 2176–2179.

3) Della Sala, P.; Del Regno R.; Talotta, C.; Capobianco, A.; Hickey, N.; Geremia, S.; De Rosa, M.; Spinella, A.; Soriente, A.; Spinella, A.; Neri, P.; Gaeta, C. Prismarenes: A New Class of Macrocyclic Hosts Obtained by Templation in a Thermodynamically Controlled Synthesis *J. Am. Chem. Soc.* **2020**, *142,* 1752–1756*.*

4) Della Sala, P.; Del Regno R.; Di Marino, L.; Calabrese, C., Palo, C.; Talotta, C.; Geremia, S.; Hickey, N.; Capobianco, A.; Neri, P.; Gaeta, C. An intramolecularly self-templated synthesis of macrocycles: self-filling effects on the formation of prismarenes *Chem. Sci.* **2021**, *12*, 9952–9961.

5) Pracht, P., Bohle, F., Grimme, S. Automated exploration of the low-energy chemical space with fast quantum chemical methods. *Phys. Chem. Chem. Phys.* **2020,** *22*, 7169-7192.

6) Bannwarth, C., Caldeweyher, E., Ehlert, S., Hansen, A., Pracht, P., Seibert, J., et al. Extended tight-binding quantum chemistry methods. WIREs Computational Molecular Science n/a, e01493.
